# Dietary intake of tomato and lycopene, blood levels of lycopene, and risk of total and specific cancers in adults: a systematic review and dose–response meta-analysis of prospective cohort studies

**DOI:** 10.3389/fnut.2025.1516048

**Published:** 2025-02-12

**Authors:** Arghavan Balali, Kimia Fathzadeh, Gholamreza Askari, Omid Sadeghi

**Affiliations:** ^1^Nutrition and Food Security Research Center, Department of Community Nutrition, School of Nutrition and Food Science, Isfahan University of Medical Sciences, Isfahan, Iran; ^2^Department of Community Nutrition, School of Nutritional Sciences and Dietetics, Tehran University of Medical Sciences, Tehran, Iran; ^3^Research Center for Food Hygiene and Safety, School of Public Health, Shahid Sadoughi University of Medical Sciences, Yazd, Iran

**Keywords:** LYCOPENE, tomato, Cancer, mortality, meta-analysis

## Abstract

**Background:**

The association between tomato/lycopene intake and blood levels of lycopene with the risk of specific cancers were assessed in previous meta-analyses; however, no study evaluated the risk of overall cancer incidence/mortality. Therefore, the present systematic review and dose–response meta-analysis aimed to summarize available findings from prospective studies to examine the association between tomato/lycopene intake and lycopene levels with the risk of total and specific cancers and cancer-related mortality.

**Methods:**

A comprehensive literature search was done using Scopus, PubMed, ISI Web of Science, and Google Scholar until July 2023.

**Results:**

In total, 121 prospective studies were included in the systematic review and 119 in the meta-analysis. During the follow-up period of 2–32 years, a total of 108,574 cancer cases and 10,375 deaths occurred. High intakes and high levels of lycopene compared to low amounts were, respectively, associated with 5% (Pooled RR: 0.95, 95% CI: 0.92–0.98, I^2^ = 26.4%, *p* = 0.002) and 11% (Pooled RR: 0.89, 95% CI: 0.84–0.95, I^2^ = 15.0%, *p* < 0.001) reduction in overall cancer risk. Also, each 10 μg/dL increase in blood levels of lycopene was associated with a 5% lower risk of overall cancer. Moreover, we found a linear inverse association between dietary lycopene intake and prostate cancer risk (Pooled RR 0.99, 95% CI 0.97–1.00, I^2^ = 0, *p* = 0.045). Regarding cancer mortality, negative relationships were found with total tomato intake (Pooled RR: 0.89, 95% CI: 0.85–0.93, I^2^ = 65.7%, *p* < 0.001), lycopene intake (Pooled RR: 0.84, 95% CI: 0.81–0.86, I^2^ = 86.5%, *p* < 0.001) and lycopene levels (Pooled RR 0.76, 95% CI: 0.60–0.98, I^2^ = 70.9%, *p* = 0.031). Also, an inverse association was observed between blood lycopene levels and lung cancer mortality (Pooled RR: 0.65, 95% CI: 0.45–0.94, I^2^ = 0, *p* = 0.022).

**Conclusion:**

Our findings show that dietary intake and blood levels of lycopene are associated with a lower risk of cancer and death due to cancer.

**Clinical trial registration:**

CRD42023432400.

## Introduction

Diet has a potential role in the etiology of cancer, therefore dietary factors are responsible for 5–10% of cancer incidence (1–3). Based on the current evidence, fruit and vegetable intake may protect against cancer incidence and mortality (4, 5). The nutrient content of these food groups, such as fiber, vitamin C, and other antioxidants such as carotenoids and polyphenols, might explain the protective effect. Recently, the association between tomato intake and cancer risk received significant attention. Tomato contains different carotenoids, including *β*-carotene, lycopene, and lutein. Lycopene is a 40-carbon red pigment with antioxidant properties that is extracted from watermelon, apricot, and other red fruits and vegetables in addition to tomatoes. However, it is estimated that more than 80% of lycopene intake is from tomatoes and their products.

Several studies have shown that tomato intake is associated with a reduced risk of cancer and cancer progression. However, it is not clear that the beneficial effect is medicated by lycopene or other nutrients available in tomatoes. Experimental studies revealed that lycopene may have anticancer properties by regulating gene expression, modulating hormone and immune activity, and also stimulating the clearance of carcinogens (3). Despite the mentioned mechanisms, findings from observational studies on the associations of tomato and lycopene with cancer risk and mortality are conflicting (6–136). Some studies reported that dietary intake of lycopene or tomato was inversely associated with cancer risk (11, 13, 14, 42), while other studies indicated this inverse association for tomato or lycopene only. Also, there are inconsistent results between dietary and serum levels of lycopene in relation to cancer risk. In addition, a large number of studies found a null association between tomato and lycopene intake and risk of cancer incidence/mortality.

Although there are several meta-analyses in this area, we found no meta-analysis that considered all the exposures (tomato intake, dietary and blood levels of lycopene) together and the risk of cancer incidence/mortality. We found four meta-analyses on prostate cancer (137–140), one for pancreatic cancer (141), one for gastric cancer (142), two for breast cancer (143, 144), and one for ovarian cancer (145). It should be noted that findings from these meta-analyses are inconsistent, and there is no summary evidence for other types of cancers. Therefore, performing a meta-analysis considering all types of cancers is necessary. In addition, the dose–response analyses were not determined in some previous meta-analyses. Taken together, the current systematic review and dose–response meta-analysis were done to determine the associations of tomato intake and dietary/blood levels of lycopene with the risk of total and specific cancers and their mortality by summarizing available findings from prospective cohort studies.

## Methods

The current study was performed in accordance with the Preferred Reporting Items for Systematic Reviews and Meta-analysis (PRISMA) (146). The protocol for this systematic review was registered on PROSPERO with the code CRD42023432400.

### Data source and search strategy

We searched databases, including PubMed, Scopus, and Web of Science, up to July 2023 to identify prospective studies that examined the association between dietary intake of tomato and lycopene and blood levels of lycopene with the risk of total and specific cancers or their mortality. The terms used in the search strategy are presented in Supplementary Table 1. No restrictions in language and time were considered. All results were included in Endnote software, and duplicate papers were removed. Eligible publications were selected based on the inclusion and exclusion criteria by two investigators (AB and KF). To maximize the search, we reviewed reference lists of selected articles and also previous systematic reviews. In addition, a manual search was done in Google Scholar using “tomato” and “lycopene” keywords separately with “cancer” to find any missing articles. The first 300 relevancy-ranked papers of this search engine were screened.

#### Inclusion criteria

Articles were considered for inclusion if they (1) were prospective in design, (2) evaluated the association between dietary intake of lycopene or tomato, or blood levels of lycopene with risk of cancer or cancer-related mortality, (3) were performed on adults (≥ 18 y), (4) those studies that reported odds ratio (OR) or risk ratio (RR) or hazard ratio (HR) along with 95% confidence intervals (CIs) for the association between tomato/lycopene and cancer risk and mortality. If the results of 1 dataset were published in >1 article, we chose the one with the most significant number of cases or more extended follow-up period.

#### Exclusion criteria

We excluded studies if they were case–control or cross-sectional in design, letters, review articles, editorials, and poster abstracts. Moreover, studies that investigated the combination association of tomatoes and other vegetables with cancer were excluded. In addition, studies with insufficient data and those that were done on critically ill patients were not included. Moreover, we excluded studies that evaluated lycopene supplementation in relation to cancer risk. Those studies that considered specific types of tomato products, such as tomato sauce, rather than raw tomato or total tomato intake, were excluded as well.

### Data extraction

Two independent reviewers (AB and KF) extracted the following data from each eligible study: first author’s name, year of publication, country, participant’s age and gender, sample size, follow-up duration, cohort name, methods used for assessment of exposures (tomato and lycopene intake and blood levels of lycopene) and outcome (cancer incidence), covariates used for adjustment, and any reported effect sizes (ES) and corresponding 95% CIs for the association between dietary intake of tomato/lycopene with risk of total and specific cancers and their mortality.

### Quality assessment

Two researchers (AB and KF) independently assessed the quality of all included studies using the Newcastle Ottawa Scale (NOS) (147). According to this scale, a maximum of 9 points would be awarded to each study according to the following parameters: 4 points for selection of participants, 2 points for comparability, and 3 points for the assessment of outcomes. Studies achieving a total score of ≥7 (median score of studies included in the current meta-analysis) were considered high-quality studies.

### Statistical analysis

We included the RR of cancer and cancer mortality reported for the comparison between the highest and lowest intakes of lycopene and tomato and the highest and lowest circulating levels of lycopene in the meta-analysis. However, some studies reported RRs of cancer risk per 1 standard deviation (SD) increment in exposure levels. To include such studies in the meta-analysis, we converted the per SD increment risk estimates to the relative risks for the comparison of the top versus bottom quartile using the method suggested by Danesh et al. (148) in which the log risk estimates reported for the comparison are equivalent to 2.54 times the log risk estimates for a 1 SD increase. This method assumes that the exposure is a normally distributed variable and that the association with the disease risk is log-linear. Moreover, in the populations where the prevalence of cancer was ≥10%, we converted reported ORs and HRs to RRs before meta-analysis.

Since the between-study heterogeneity was low in most analyses, we used a fixed-effects model to calculate the overall effect estimates of cancer risk and mortality. In addition to the fixed model, we performed the overall analyses using a random-effects model. This model considers different sources of uncertainties, including within- (sampling or estimation) and between-studies heterogeneity (149). However, since random-effects models tend to give disproportionally more weight to smaller studies, mainly when the outcome is binary (e.g., cancer or death), fixed-effects models may present more reliable results compared with the random-effects models (150). Cochran’s *Q* test and the I^2^ statistic were used to assess heterogeneity among included studies. I^2^ values of >50%, or *p* < 0.10 for the *Q*-test, were considered as significant heterogeneity. To identify possible sources of heterogeneity, subgroup analyses were performed based on pre-defined variables including duration of follow-up (≥10 vs. <10 years), sample size (≥10,000 vs. <10,000 participants), geographical location (US vs. non-US countries), methods used for the assessment of exposures (FFQ vs. other tools) and outcome (medical records or pathological methods vs. self-reported data), study quality (high vs. low), adjustments for important confounders including energy intake and BMI (adjusted vs. not-adjusted), and tissue levels of lycopene (serum vs. plasma). We selected the variables based on their effects on the findings of our meta-analysis (i.e., follow-up duration, sample size, etc.) and the importance of results in their subgroups (i.e., geographical location, study quality, etc.). We used the formal tests of Egger and Begg to detect potential publication bias. Moreover, a sensitivity analysis using a random-effects model was performed to examine the dependency of overall risk estimates on each study.

In addition to the highest versus lowest comparison, we assessed the linear and non-linear dose–response associations between tomato/lycopene intakes, serum levels of lycopene, and cancer risk. For the linear dose–response analysis, the generalized least squares trend (GLST) estimation method, described by Greenland and Longnecker (151) and Orsini et al. (152), was used. First, we estimated study-specific slopes, and then these slopes were combined to obtain an overall average slope. We combined the study-specific slopes using random- or fixed-effects models. In the GLST method, the distribution of cases, the total number of participants, and the effect sizes with the variance estimates for ≥3 quantitative categories of exposure were required. The following information was required in this method for each study: distribution of total participants and cancer cases, RRs of cancer risk or mortality across categories of exposures, and the median or mean amount of serum or dietary tomato/lycopene in each category. In studies that reported the amount of exposure as ranges in each category, we estimated the midpoint by calculating the mean of the lower and upper bound. For open-ended categories, we considered the length of the category the same as an adjacent interval. For studies with reported raw tomato consumption as serving/day, we converted it to gr/day using the serving size (in grams) presented in the studies. For studies that did not report the amount of serving size, the standard serving size of 180 grams was used for this conversion. The non-linear dose–response relationship was also assessed using the restricted cubic splines with 3 knots at percentiles of 10, 50, and 90% of the distribution. The correlation within each set of provided risk estimates was considered, and the study-specific estimates were combined using a one-stage linear mixed-effects meta-analysis. The significance for nonlinearity was calculated by null hypothesis testing, in which the coefficient of the second spline was considered equal to zero. All statistical analyses were done using Stata software, version 17 (Stata Corp, College Station, TX). *p*-values were considered significant at the level of <0.05.

## Results

### Search results

**Figure 1:**
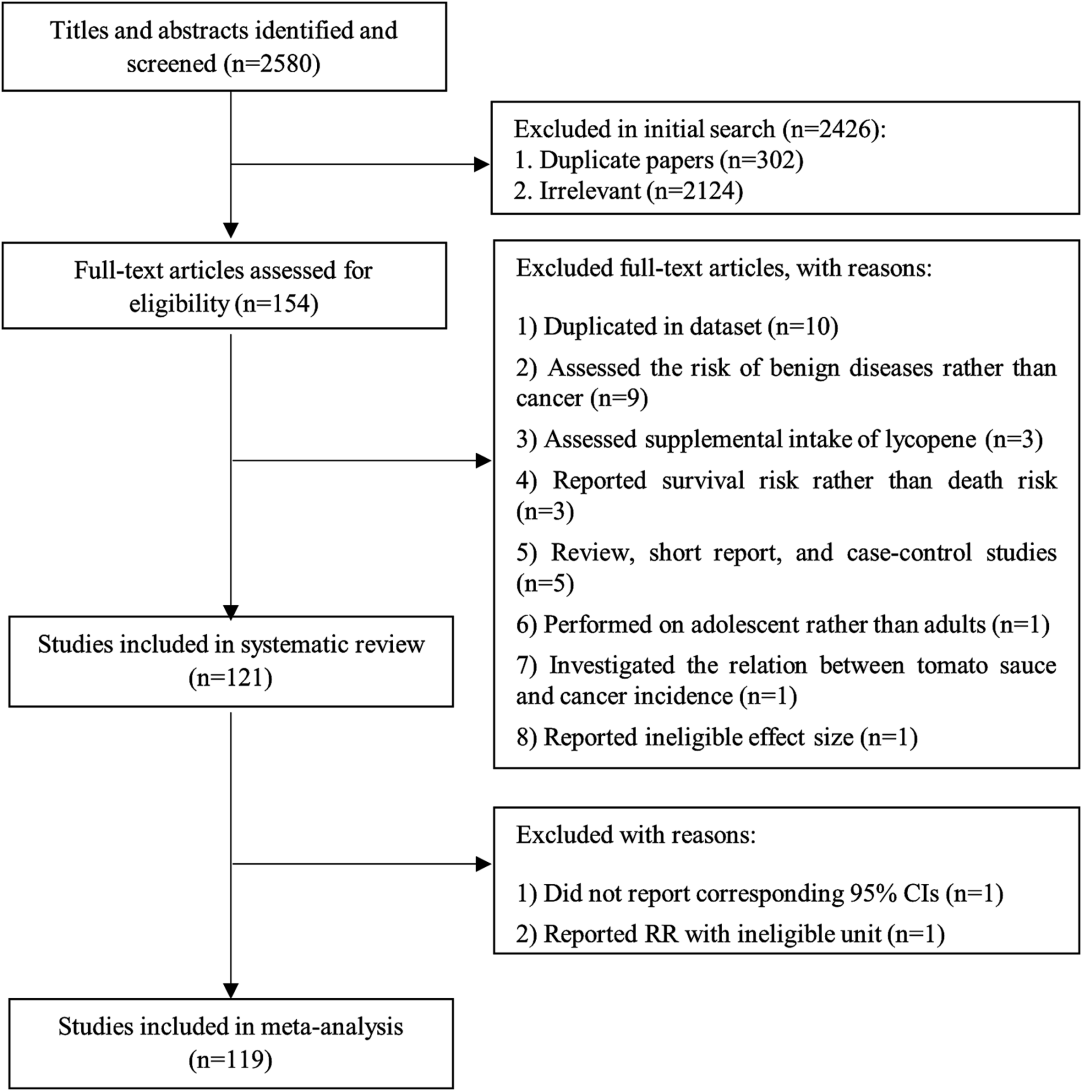
Flow diagram of study selection.

We found a total of 2,580 papers in the online databases. After excluding duplicate papers (*n* = 302), 2,278 articles remained for the title and abstract review. Accordingly, 2,124 papers were considered unrelated, and 154 articles were included in the full-text assessment. Of the 154 articles, nine studies were excluded because the risk of benign diseases was assessed rather than cancer risk (153–161). We also excluded three studies that reported survival risk instead of death risk (111, 162, 163). Three studies assessed the lycopene supplementation in relation to cancer risk and, therefore, were excluded (164–166). One study performed on children and adolescents was excluded as well (167). Moreover, in one study, the relationship between the consumption of tomato sauce and cancer incidence was investigated. Therefore, it was excluded (168). In addition, one study was excluded because of reporting correlation coefficient rather than RR (169). Furthermore, three studies with a case–control design (170–172), one review article (173), and one short report (174) were excluded. We found three publications which were conducted on Health Professional Follow-up Study (HPFS) (37, 128, 129), four papers on Nurses’ Health Study (NHS) (76, 130–132), two papers on Comprehensive Health Examination Program (CHEP) (113, 133), two articles on Japan Collaborative Cohort Study (JACC) (105, 134), two publications on the Prostate, Lung, Colorectal, and Ovarian trial (PLCO) (17, 135), and two on The European Prospective Investigation into Cancer and Nutrition (EPIC) (70, 136). With respect to these articles assessed similar exposure and outcome variables, we included only the one with the highest quality or with the most significant number of cases for each dataset (17, 37, 70, 76, 105, 113) and excluded the duplicated papers (128–136). Also, we found a pooled analysis of 10 datasets (121). All studies included in the pooled analysis, except the data from the NHS (9), were different. To avoid double-counting data, we excluded the study of Fairfield et al. (9) containing data from the NHS, and therefore, we included the pooled analysis. Finally, after these exclusions, 121 articles containing prospective studies were included in the current systematic review. Figure 1 summarizes the process of study selection.

### Overview of the included studies

Supplementary Tables 2, 3 illustrate the characteristics of included studies in the current systematic review and meta-analysis. The sample size of included studies ranged between 102 and 521,911 participants, resulting in a total sample size of 4,598,358 subjects aged 18–104 years. During follow-up periods ranging from 2 to 32 years, a total of 108,574 cancer cases and 10,375 deaths due to cancer were recognized. Out of 121 articles, 66 were conducted in the US, 54 in non-US countries, and one in both US and non-US countries. Dietary intakes of lycopene and tomato were assessed using FFQ in 59 articles, a researcher-made questionnaire in 3 publications, dietary history in 3 papers, food recall in 2 papers, food record in 2 papers, and both researcher-made questionnaire and food recall in one study. In terms of cancer assessment, 28 papers used self-reported data, 84 articles used data from medical records, two papers used both medical records and self-reported data, and other studies used pathological or histological findings for cancer diagnosis. Among the included studies, different confounding variables, including energy intake (n = 52), BMI, smoking, and age, were adjusted. The NOS scores of the included studies ranged between 5 and 9. We considered the score of 7 as the median for a total score of NOS; 88 articles had a score of ≥7, defined as high-quality studies (Supplementary Tables 4, 5).

### Findings from the systematic review

From 46 articles on dietary lycopene and overall cancer risk, four papers found an inverse association, and others illustrated no significant association. Of the 19 papers on total tomato consumption and overall cancer risk, four indicated an inverse association, but others found no significant association. Two articles illustrated an inverse association between blood levels of lycopene and total cancer risk (*n* = 43). In the case of cancer mortality, one study indicated a protective association between lycopene intake and cancer mortality. Such a protective association was also found in two studies for total tomato intake and three papers for blood levels of lycopene; however, others showed no significant association.

### Findings from the meta-analysis

Of the 121 articles in the systematic review, 119 papers with complete data were included in the current meta-analysis. One study that reported RRs without corresponding 95% CIs was not included (127). Moreover, the study of Fujii et al. (112) was excluded because they reported an RR of cancer mortality per 25% increase in serum lycopene. Since the conversion of this unit to other usual units was impossible, we excluded this study from the meta-analysis. Some included papers reported RRs of different cancers from one dataset. To avoid double-counting data, we first merged the RRs to calculate an overall RR of cancer for that dataset. Then, the pooled RR was included in the primary meta-analysis. Accordingly, we merged the effect sizes of 3 papers from the NHS (8, 15, 36), five papers from the Women’s Health Initiative Study (WHI) (7, 13, 32, 108, 109), five publications from the Alpha-Tocopherol, Beta-Carotene Cancer Prevention Study (ATBC) (14, 20, 23, 26, 123), two papers from the PLCO (17, 33), two papers from the HPFS (21, 37), two articles from the Multiethnic Cohort Study (MEC) (28, 29), six articles from the Netherlands Cohort Study (NLCS) (30, 55, 56, 64, 65, 125), three papers from the NLCS (67, 68, 120), five publications from the National Breast Screening Study (NBSS) (24, 57, 59, 61, 62), two papers from the Singapore Chinese Health Study (SCHS) (35, 63), and two articles from both NHS and HPFS (16, 22) to calculate overall RRs of cancer.

### Total tomato intake and cancer

#### Overall cancer

Nineteen papers (11, 17, 31, 40, 42–49, 52, 60, 63, 66, 67, 69, 124) with a total of 1,120,154 participants and 30,009 cases were included in this association. Overall RR for this relation, comparing the highest with the lowest intake of tomato, was 1.01 (95% CI: 0.97–1.05, *p* = 0.687), indicating no significant association between total tomato intake and overall risk of cancer (Table 1). Also, there was evidence of significant heterogeneity between the studies (I^2^ = 61.0%, *p* < 0.001).

**Table 1:** Summary risk estimates for the association between tomato intake with cancer risk and mortality in adults.

	*n* ^1^	Pooled RR (95% CI)^2^	P^3^	I^2^ (%)^4^	P-heterogeneity	P-interaction
The highest vs. lowest comparison of total tomato intake						
Overall cancer risk	19	1.01 (0.97–1.05)	0.687	61.0	<0.001	
Subgroup analyses						
Study location						0.719
US	11	1.02 (0.96–1.07)	0.574	61.8	0.005	
Non-US	8	1.00 (0.93–1.07)	0.973	68.8	0.002	
Sample size						0.056
≥ 10,000 participants	15	1.00 (0.96–1.05)	0.994	67.7	<0.001	
< 10,000 participants	4	1.22 (1.00–1.49)	0.050	0	0.530	
Adjustment for energy						0.558
Yes	14	1.00 (0.95–1.05)	0.913	68.3	<0.001	
No	5	1.03 (0.94–1.13)	0.472	50.0	0.092	
Adjustment for BMI						0.002
Yes	14	1.04 (0.99–1.08)	0.142	60.0	0.003	
No	5	0.83 (0.73–0.95)	0.005	35.6	0.184	
Quality of studies						0.354
High quality	13	1.02 (0.97–1.07)	0.402	67.4	<0.001	
Low quality	6	0.97 (0.89–1.06)	0.558	56.7	0.042	
Follow-up duration						0.322
≥ 10 years	9	1.02 (0.97–1.08)	0.364	72.5	<0.001	
< 10 years	10	0.98 (0.90–1.06)	0.557	50.1	0.042	
Dietary intake assessment						0.012
FFQ	15	0.95 (0.89–1.01)	0.128	59.9	0.002	
Others	4	1.06 (1.00–1.13)	0.042	59.8	0.059	
Cancer assessment						0.037
Medical reports or pathological methods	13	0.95 (0.89–1.02)	0.170	64.2	0.001	
Self-reported	6	1.05 (0.99–1.11)	0.104	54.7	0.050	
Specific cancers						
Breast	5	1.04 (0.97–1.10)	0.286	46.0	0.116	
Prostate	8	1.02 (0.96–1.09)	0.482	57.4	0.022	
Overall cancer mortality	4	0.89 (0.85–0.93)	<0.001	65.7	0.033	
Linear dose–response association (per 50-g/d increase)						
Overall cancer	10	1.00 (0.97–1.02)	0.779	55.4	0.017	
Subgroup analyses						
Study location						0.369
US	2	0.99 (0.95–1.02)	0.430	0	0.445	
Non-US	8	1.01 (0.97–1.05)	0.608	62.7	0.009	
Sample size						0.071
≥ 10,000 participants	8	0.99 (0.96–1.02)	0.400	58.7	0.018	
< 10,000 participants	2	1.07 (0.99–1.16)	0.105	0	0.999	
Adjustment for energy						0.044
Yes	8	0.99 (0.96–1.01)	0.298	56.5	0.024	
No	2	1.06 (0.99–1.14)	0.080	0	0.912	
Adjustment for BMI						0.244
Yes	7	1.00 (0.98–1.03)	0.791	63.2	0.012	
No	3	0.97 (0.91–1.02)	0.242	21.0	0.282	
Quality of studies						0.044
High quality	8	0.99 (0.96–1.02)	0.298	56.5	0.024	
Low quality	2	1.06 (0.99–1.14)	0.080	0	0.912	
Follow-up duration						0.460
≥ 10 years	5	0.98 (0.93–1.03)	0.434	73.9	0.004	
< 10 years	5	1.00 (0.97–1.03)	0.906	7.8	0.362	
Dietary intake assessment						0.138
FFQ	9	0.99 (0.96–1.02)	0.505	55.5	0.021	
Others	1	1.07 (0.97–1.18)	0.176	0	0	
Cancer assessment						0.136
Medical reports or pathological methods	8	0.99 (0.96–1.02)	0.480	60.8	0.013	
Self-reported	2	1.06 (0.97–1.16)	0.179	0	0.750	
Specific cancers						
Prostate	3	1.01 (0.98–1.04)	0.588	51.6	0.127	
The highest vs. lowest comparison of raw tomato intake						
Overall cancer risk	5	1.03 (0.96–1.11)	0.396	26.7	0.243	
Specific cancers						
Prostate	4	1.05 (0.97–1.15)	0.247	0	0.994	
Linear dose–response association (per 50-g/d increase)						
Overall cancer risk	5	0.99 (0.95–1.03)	0.573	0	0.636	
Specific cancers						
Prostate	4	1.00 (0.96–1.04)	0.881	0	0.954	

n, Number, RR, Relative risk; CI, Confidence interval; US, United States; BMI, Body mass index; FFQ, Food frequency questionnaire; g, Gram; d, Day. ^1^Number of effect sizes. ^2^Obtained from the fixed-effects model. ^3^Obtained from the *Q*-test. ^4^Inconsistency – the percentage of variation across studies due to heterogeneity.

**Figure 2:**
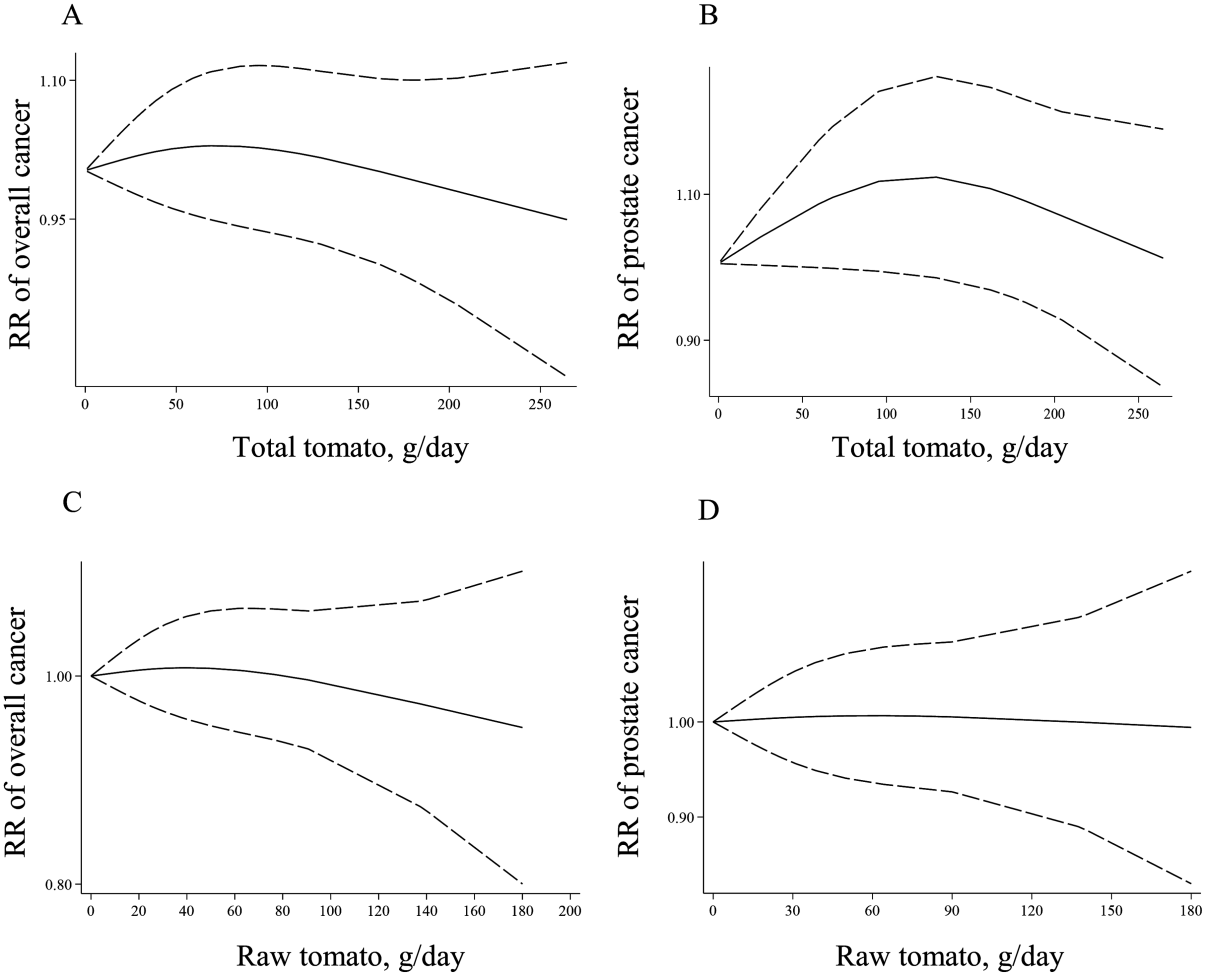
Non-linear dose–response associations of total tomato consumption with risk of overall **(A)** and prostate cancer **(B)**, and non-linear dose–response associations of raw tomato consumption with risk of overall **(C)**, and prostate cancer **(D)** in adults aged ≥18 years. The solid lines indicate the spline model. The dashed lines present the 95% CI. RR: relative risk.

Eight (17, 40, 44, 46, 48, 49, 52, 63) and 12 articles (17, 40, 41, 44, 46, 48, 49, 52, 63, 67, 68, 120) with sufficient data were recognized for inclusion in the non-linear and linear dose–response analysis, respectively. We found no significant association between a 50-g/d increase in total tomato intake and overall risk of cancer (Pooled RR: 1.00, 95% CI: 0.97–1.02, *p* = 0.779; Table 1). Moreover, there was no evidence of a non-linear association in this regard (P for nonlinearity = 0.618; Figure 2A).

#### Specific cancers

Overall, combining five articles on breast cancer (44, 48, 66, 69, 124) and eight publications on prostate cancer (11, 17, 31, 40, 45, 47, 49, 67), comparing the highest and lowest intakes of total tomato, presented an overall RR of 1.04 (95% CI: 0.97–1.10, I^2^ = 46.0%, *p* = 0.286) for breast cancer and 1.02 (95% CI: 0.96–1.09, I^2^ = 57.4%, *p* = 0.482) for prostate cancer that both were statistically non-significant (Table 1).

Regarding prostate cancer, three publications (17, 40, 49) had sufficient data to perform non-linear and linear dose–response analysis. We found no linear (Table 1) and non-linear (Figure 2B) associations for this cancer (P for nonlinearity = 0.157).

#### Cancer mortality

Four articles (38, 39, 50, 51) with a total sample size of 249,308 and 8,863 cancer deaths were included. Summary RR of cancer mortality, comparing the highest and lowest intakes of total tomato, was 0.89 (95% CI: 0.85–0.93, I^2^ = 65.7%, *p* < 0.001), indicating a significant inverse association (Table 1).

### Raw tomato intake and cancer

#### Overall cancer

Six papers (6, 10, 17, 31, 33, 44) with a total of 285,840 participants and 8,429 cases were included in this association. The summary effect size for the risk of total cancer comparing the highest with the lowest intakes of raw tomato was 1.03 (95% CI: 0.96–1.11; I^2^ = 26.7%, *p* = 0.396), indicating a non-significant positive association (Table 1).

Six articles (6, 10, 17, 31, 33, 44) with sufficient data were included in the linear and non-linear dose–response analyses. We found no linear (Table 1) and non-linear (Figure 2C) associations for the overall cancer risk (P for nonlinearity = 0.777).

#### Specific cancers

Overall, combining four articles (6, 10, 17, 31) on prostate cancer, we found no significant association when comparing the highest and the lowest categories of raw tomato intake (RR: 1.05, 95% CI: 0.97–1.15, *p* = 0.247) (Table 1).

Four studies (6, 10, 17, 31) with sufficient data were included in linear and non-linear dose–response analyses. We found no significant linear association between each 50-g/d increase in raw tomato intake and risk of prostate cancer (Table 1). Moreover, there was no evidence of non-linear association in this regard (P for nonlinearity = 0.978; Figure 2D).

### Dietary lycopene intake and cancer

#### Overall cancer

Forty-six articles (7, 8, 10, 12–30, 32, 33, 35–37, 53–66, 121–123, 125, 126) with a total of 2,687,842 subjects and 49,617 cases were included in this association. The summary effect size for the risk of total cancer comparing the highest with the lowest intakes of lycopene was 0.95 (95% CI: 0.92–0.98; I^2^ = 26.4%, *p* = 0.002), indicating a significant inverse association (Table 2).

**Table 2:** Summary risk estimates for the association between dietary intake of lycopene with cancer risk and mortality in adults.

	*n* ^1^	Pooled RR (95% CI)^2^	P^3^	I^2^ (%)^4^	P-heterogeneity	P-interaction
The highest vs. lowest comparison of lycopene consumption						
Overall cancer	24	0.95 (0.92–0.98)	0.002	26.4	0.117	
Subgroup analysis						
Study location						0.405
US	14	0.96 (0.93–1.00)	0.054	42.4	0.047	
Non-US	9	0.92 (0.86–0.98)	0.008	0	0.550	
US and Non-US	1	0.97 (0.84–1.12)	0.678	-	-	
Sample size						0.178
≥ 10,000 participants	12	0.94 (0.91–0.98)	0.001	42.4	0.042	
< 10,000 participants	9	0.99 (0.93–1.06)	0.839	0	0.742	
Adjustment for energy						0.218
Yes	17	0.94 (0.91–0.98)	0.001	38.1	0.056	
No	7	0.99 (0.92–1.07)	0.884	0	0.693	
Adjustment for BMI						0.004
Yes	16	0.99 (0.95–1.03)	0.711	3.4	0.414	
No	8	0.90 (0.86–0.95)	<0.001	2.7	0.409	
Quality of studies						0.268
High quality	17	0.94 (0.91–0.98)	0.001	36.9	0.064	
Low quality	7	0.99 (0.92–1.06)	0.759	0	0.586	
Follow-up duration						0.018
≥ 10	10	0.92 (0.88–0.96)	<0.001	34.9	0.129	
< 10	14	0.99 (0.95–1.04)	0.779	0	0.545	
Dietary intake assessment						0.220
FFQ	19	0.96 (0.93–0.99)	0.023	19.8	0.213	
Others	5	0.91 (0.84–0.98)	0.019	45.2	0.121	
Cancer assessment						0.018
Medical reports or pathological methods	19	0.98 (0.94–1.02)	0.365	9.8	0.336	
Self-reported	5	0.91 (0.86–0.96)	<0.001	30.1	0.221	
Specific cancers						
Breast	8	0.99 (0.93–1.07)	0.971	0	0.528	
Prostate	8	0.95 (0.90–1.00)	0.068	16.1	0.303	
Ovarian	3	0.97 (0.86–1.10)	0.645	0	0.923	
Lung	7	0.83 (0.75–0.92)	<0.001	27.0	0.222	
Bladder	5	1.11 (0.96–1.28)	0.171	0	0.771	
Colorectal	3	1.08 (0.94–1.23)	0.265	0	0.926	
Gastric	3	0.87 (0.67–1.12)	0.286	0	0.472	
Pancreatic	3	0.97 (0.78–1.22)	0.814	0	0.658	
Overall cancer mortality	3	0.84 (0.81–0.86)	<0.001	86.5	0.001	
Linear dose–response association (per 10-mg/d increase)						
Overall cancer	22	0.99 (0.98–1.02)	0.137	39.5	0.031	
Subgroup analysis						
Study location						0.636
US countries	12	0.99 (0.98–1.01)	0.089	38.6	0.084	
Non-US countries	9	1.00 (0.98–1.02)	0.804	49.6	0.044	
US and Non-US countries	1	1.02 (0.93–1.12)	0.690	-	-	
Sample size						0.310
≥ 10,000 participants	14	0.99 (0.97–1.00)	0.072	50.5	0.016	
< 10,000 participants	8	1.00 (0.98–1.02)	0.971	5.1	0.391	
Adjustment for energy						0.282
Yes	15	0.99 (0.98–1.01)	0.067	49.5	0.016	
No	7	1.00 (0.98–1.02)	0.953	0	0.443	
Adjustment for BMI						0.718
Yes	17	0.99 (0.98–1.02)	0.429	32.8	0.093	
No	5	0.99 (0.97–1.01)	0.190	62.7	0.030	
Quality of studies						0.306
High quality	17	0.99 (0.98–1.01)	0.071	42.9	0.031	
Low quality	5	1.00 (0.98–1.02)	0.975	28.5	0.231	
Follow-up duration						0.647
≥ 10	10	0.99 (0.97–1.00)	0.122	58.3	0.010	
< 10	12	1.00 (0.97–1.03)	0.881	14.7	0.300	
Dietary intake assessment						0.338
FFQ	17	0.99 (0.98–1.02)	0.109	19.2	0.229	
Others	5	1.03 (0.95–1.12)	0.455	71.4	0.007	
Cancer assessment						0.286
Medical reports or pathological methods	18	1.00 (0.98–1.01)	0.817	16.4	0.258	
Self-reported	4	0.99 (0.97–1.00)	0.070	77.3	0.004	
Specific cancers						
Breast	7	1.00 (0.98–1.02)	0.815	0	0.468	
Prostate	7	0.99 (0.97–1.00)	0.045	0	0.582	
Lung	5	0.91 (0.72–1.14)	0.398	81.3	<0.001	
Bladder	5	1.01 (0.88–1.16)	0.835	18.5	0.297	
Colorectal	3	1.01 (0.86–1.17)	0.942	24.6	0.265	
Gastric	3	0.96 (0.34–2.72)	0.941	25.0	0.264	
Pancreatic	3	0.99 (0.96–1.03)	0.618	0	0.488	
Overall cancer mortality	3	0.85 (0.82–0.87)	<0.001	94.6	<0.001	

n, Number; RR, Relative risk; CI, Confidence interval; US, United States; BMI, Body mass index; FFQ, Food frequency questionnaire; mg, Milligram; d, Day. ^1^Number of effect sizes. ^2^Obtained from the fixed-effects model. ^3^Obtained from the *Q*-test. ^4^Inconsistency – the percentage of variation across studies due to heterogeneity.

**Figure 3:**
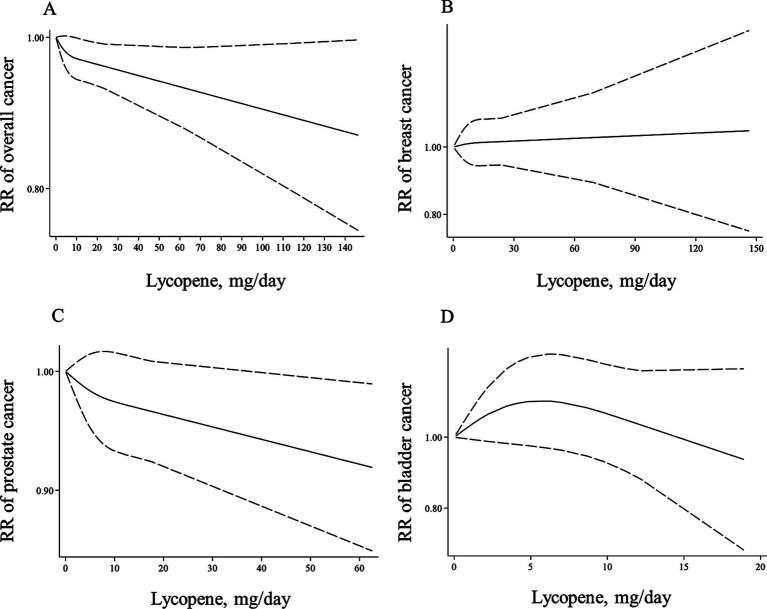
Non-linear dose–response associations of lycopene consumption with risk of overall **(A)**, breast **(B)**, prostate **(C)**, and bladder cancer **(D)** in adults aged ≥18 years. The solid lines indicate the spline model. The dashed lines present the 95% CI. RR: relative risk.

Forty-one (7, 8, 12–21, 23–30, 32, 33, 35, 37, 53–59, 61–66, 122, 123, 125, 126) and 42 papers (7, 8, 12–21, 23–30, 32, 33, 35, 37, 53–59, 61–66, 121–123, 125, 126) with sufficient data were included in the non-linear and linear dose–response analyses, respectively. There was no significant association between a 10-mg/d increase in lycopene intake and overall risk of cancer (Pooled RR: 0.99, 95% CI: 0.98–1.02, I^2^ = 39.5%, *p* = 0.137; Table 2). Moreover, the non-linear dose–response analysis indicated no non-linear relation between dietary lycopene intake and overall risk of cancer (P for nonlinearity = 0.166; Figure 3A).

#### Specific cancers

Combing eight studies for breast cancer (7, 19, 27, 36, 61, 66, 122, 126), eight studies for prostate cancer (10, 17, 29, 30, 37, 53, 54, 58), and three studies for ovarian cancer (24, 32, 121), we found no significant association when comparing the highest and the lowest categories of lycopene intake (RR for breast cancer: 0.99, 95% CI: 0.93–1.07, *p* = 0.971, RR for prostate cancer: 0.95, 95% CI: 0.90–1.00, *p* = 0.068, and RR for ovarian cancer: 0.97, 95% CI: 0.86–1.10, *p* = 0.645) (Table 2). In addition, we found no significant association for bladder, colorectal, gastric, and pancreatic cancers. However, a significant inverse association was observed between lycopene intake and risk of lung cancer (Pooled RR: 0.83, 95% CI: 0.75–0.92, I^2^ = 27.0%, *p* < 0.001) (14, 22, 25, 35, 59, 60, 64) (Table 2).

**Figure 4:**
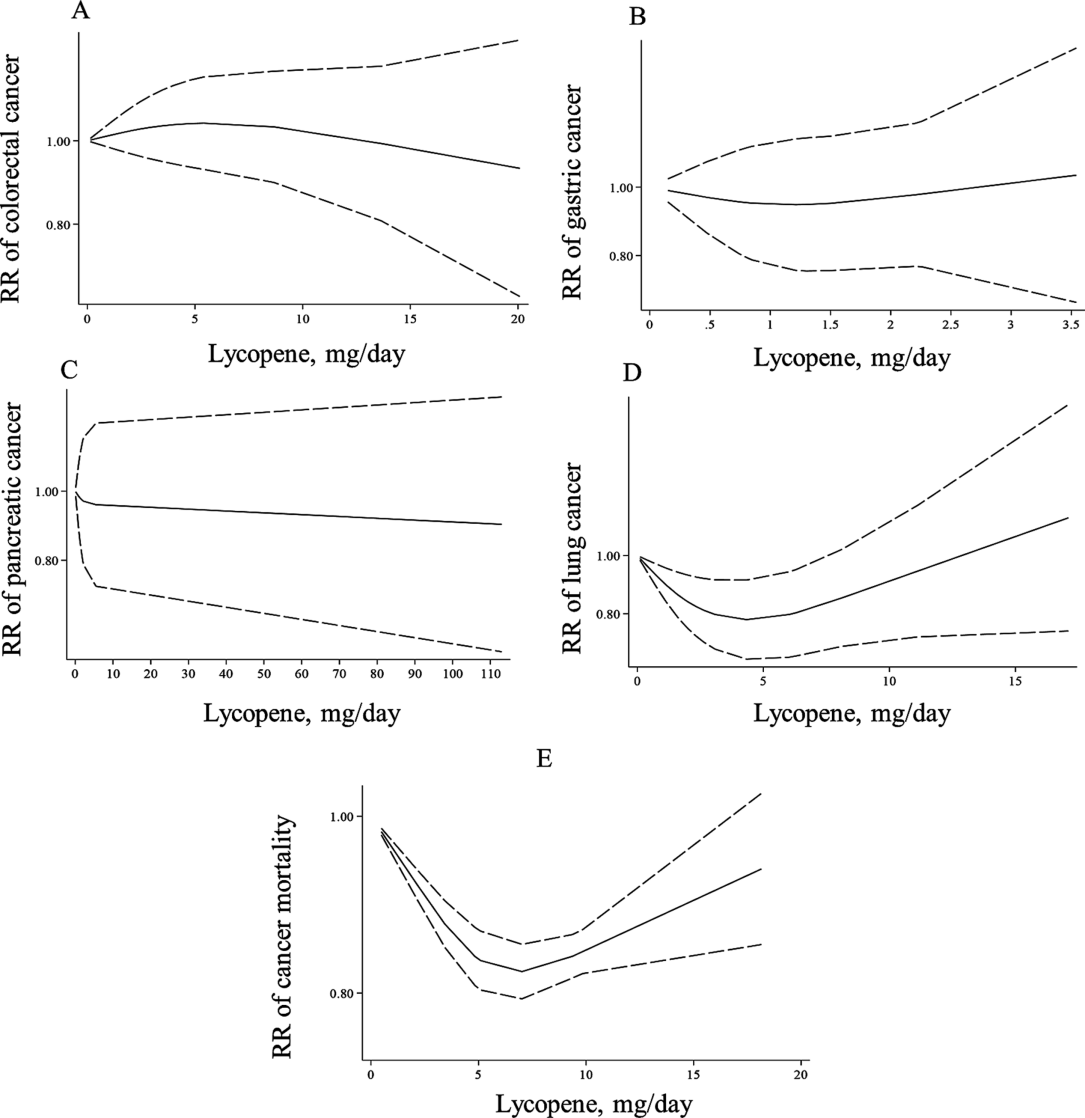
Non-linear dose–response associations of lycopene consumption with risk of colorectal **(A)**, gastric **(B)**, pancreatic **(C)**, lung cancer **(D)**, and cancer mortality **(E)** in adults aged ≥18 years. The solid lines indicate the spline model. The dashed lines present the 95% CI. RR: relative risk.

In the case of dose–response analysis, except for prostate cancer (Pooled RR: 0.99, 95% CI: 0.97–1.00, I^2^ = 0, *p* = 0.045; Table 2), we found no significant linear association between dietary lycopene intake and risk of breast, lung, bladder, colorectal, gastric, and pancreatic cancers (Table 2). Also, there was no evidence of a non-linear association for these cancers except for lung cancer, where a non-linear association was found (Figures 3B–D, 4A–C). For this association, the risk of lung cancer decreased from zero to 5 mg/d of lycopene intake, whereas the risk started to rise at approximately 10 mg/d intake (Figure 4D).

#### Cancer mortality

The summary RR for cancer mortality risk when comparing the highest with the lowest lycopene consumption was 0.84 (95% CI: 0.81–0.86, *p* < 0.001), which indicates a significant inverse association (34, 38, 39). Moreover, a significant between-study heterogeneity was observed (I^2^ = 86.5%, *p* = 0.001; Table 2). Also, there was evidence of linear dose–response association in which a 10-mg/d increase in lycopene intake was associated with a 15% risk reduction in cancer mortality (Pooled RR: 0.85, 95% CI: 0.82–0.87, I^2^ = 94.6%, p < 0.001; Table 2). Moreover, a non-linear relation was observed in this regard (P for nonlinearity <0.001; Figure 4E) in such a way that the mortality risk reduced from zero to a lycopene intake of 7 mg/d; nonetheless, the risk began to rise at a dosage of 10 mg/d.

### Blood levels of lycopene and cancer

#### Overall cancer

Forty-three articles (66, 71–103, 106–110, 117–119) with a total number of 92,356 subjects and 21,707 cases that examined the association between blood levels of lycopene and cancer risk were included in the meta-analysis. The summary RR for the risk of total cancer comparing the highest with the lowest levels of lycopene was 0.89 (95% CI: 0.84–0.95, *p* < 0.001), indicating a significant inverse association with no evidence of significant heterogeneity between studies (I^2^ = 15.0%, *p* = 0.204; Table 3).

**Table 3:** Summary risk estimates for the association between blood levels of lycopene with cancer risk and mortality in adults.

	*n* ^1^	Pooled RR (95% CI)^2^	P^3^	I^2^ (%)^4^	P-heterogeneity	P-interaction
The highest vs. lowest comparison of serum lycopene						
Overall cancer risk	42	0.89 (0.84–0.95)	<0.001	15.0	0.204	
Subgroup analysis						
Study location						0.461
US	28	0.88 (0.82–0.94)	<0.001	24.1	0.125	
Non-US	14	0.93 (0.82–1.04)	0.199	0	0.520	
Adjustment for energy						0.111
Yes	5	1.03 (0.85–1.25)	0.732	47.4	0.107	
No	37	0.88 (0.82–0.93)	<0.001	5.5	0.375	
Adjustment for BMI						0.835
Yes	22	0.89 (0.83–0.95)	0.001	17.2	0.232	
No	20	0.90 (0.80–1.01)	0.073	16.8	0.245	
Quality of studies						0.061
High quality	30	0.85 (0.79–0.92)	<0.001	12.1	0.278	
Low quality	12	0.96 (0.87–1.06)	0.401	6.1	0.386	
Follow-up duration						0.037
≥ 10	23	0.84 (0.78–0.91)	<0.001	1.1	0.446	
< 10	19	0.96 (0.87–1.05)	0.346	16.9	0.248	
Lycopene assessment						0.511
Serum levels	22	0.91 (0.83–1.00)	0.051	21.5	0.179	
Plasma levels	20	0.88 (0.81–0.95)	0.001	9.7	0.335	
Cancer assessment						0.213
Medical reports or pathological methods	34	0.87 (0.81–0.93)	<0.001	3.7	0.406	
Self-reported	9	0.94 (0.85–1.05)	0.310	43.6	0.088	
Specific cancers						
Breast	13	0.86 (0.78–0.95)	0.002	0	0.529	
Prostate	14	0.89 (0.80–0.98)	0.023	18.0	0.257	
Gastric	3	0.79 (0.59–1.06)	0.121	19.6	0.288	
Overall cancer mortality	4	0.76 (0.60–0.98)	0.031	70.9	0.016	
Specific cancers						
Lung cancer mortality	3	0.65 (0.45–0.94)	0.022	0	0.508	
Linear dose–response association (per 10-μg/dL increase)						
Overall cancer risk	34	0.95 (0.93–0.96)	<0.001	99.2	<0.001	
Subgroup analysis						
Study location						0.008
US	21	0.94 (0.93–0.96)	<0.001	99.5	<0.001	
Non-US	13	0.98 (0.95–1.02)	0.329	21.9	0.223	
Adjustment for energy						0.002
Yes	5	1.01 (0.97–1.05)	0.783	32.2	0.207	
No	29	0.94 (0.93–0.96)	<0.001	99.3	<0.001	
Adjustment for BMI						<0.001
Yes	19	1.00 (0.98–1.00)	0.005	18.0	0.235	
No	15	0.90 (0.89–0.92)	<0.001	99.4	<0.001	
Quality of studies						<0.001
High quality	26	0.94 (0.93–0.96)	<0.001	99.4	<0.001	
Low quality	8	1.00 (0.98–1.02)	0.817	0	0.686	
Follow-up duration						<0.001
≥ 10	19	0.93 (0.92–0.95)	<0.001	99.5	<0.001	
< 10	15	0.99 (0.97–1.00)	<0.001	1.4	0.435	
Lycopene assessment						<0.001
Serum levels	18	0.90 (0.89–0.92)	<0.001	99.3	<0.001	
Plasma levels	16	0.99 (0.97–1.00)	0.012	0	0.467	
Cancer assessment						<0.001
Medical reports or pathological methods	28	0.94 (0.93–0.96)	<0.001	99.3	< 0.001	
Self-reported	6	1.00 (0.98–1.02)	0.826	25.5	0.243	
Specific cancers						
Breast	12	0.98 (0.96–1.01)	0.225	21.2	0.235	
Prostate	10	0.99 (0.98–1.01)	0.485	2.3	0.418	
Gastric	3	0.92 (0.85–1.01)	0.083	0	0.390	

n, Number; RR, Relative risk; CI, Confidence interval; US, United States; BMI, Body mass index; μg, Microgram; dL, Deciliter. ^1^Number of effect sizes. ^2^Obtained from the fixed-effects model. ^3^Obtained from the *Q*-test. ^4^Inconsistency – the percentage of variation across studies due to heterogeneity.

**Figure 5:**
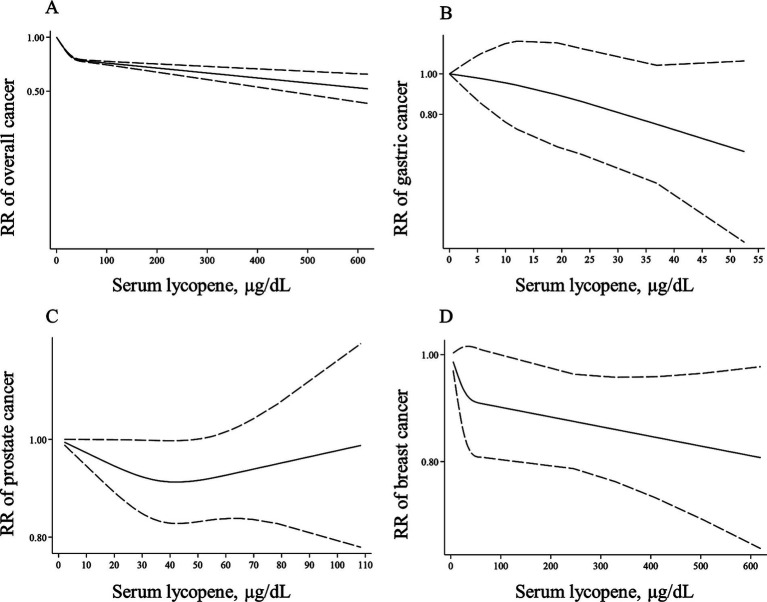
Non-linear dose–response associations of lycopene levels with risk of overall **(A)**, gastric **(B)**, prostate **(C)**, and breast cancer **(D)** in adults aged ≥18 years. The solid lines indicate the spline model. The dashed lines present the 95% CI. RR: relative risk.

Thirty-four (66, 70–72, 74–87, 89, 91, 94–99, 101, 102, 107–110, 118, 119) and 35 papers (66, 70–72, 74–87, 89, 91, 94–99, 101, 102, 107–110, 117–119) with sufficient data were included in the non-linear and linear dose–response analyses, respectively. In the linear analysis, each 10 μg/dL increase in blood levels of lycopene was associated with a 5% lower risk of total cancer (Pooled RR: 0.95, 95% CI: 0.93–0.96, *p* < 0.001; Table 3). Also, we found evidence of a non-linear association in this regard (P for nonlinearity <0.001), in which the risk of total cancer decreased continuously until 50 μg/dL of lycopene levels, and then, the risk reduction slowed down at the higher dosages (Figure 5A).

#### Specific cancers

Blood levels of lycopene in relation to breast cancer were examined in 13 studies (66, 70, 74–76, 84, 93, 97–99, 106, 108, 118) with 24,599 participants and 9,061 cases. A significant inverse association was found when comparing the highest with the lowest levels of lycopene (Pooled RR: 0.86, 95% CI: 0.78–0.95, I^2^ = 0%, *p* = 0.002). In terms of prostate cancer, we also observed a significant inverse association by comparing the highest with the lowest lycopene concentrations (Pooled RR: 0.89, 95% CI: 0.80–0.98, *p* = 0.023) with no evidence of heterogeneity between studies (I^2^ = 18.0%, *p* = 0.257; Table 3). Additionally, by comparing the highest vs. lowest lycopene concentrations, no significant association was found regarding gastric cancer (Pooled RR: 0.79, 95% CI: 0.59–1.06, I^2^ = 19.6%, *p* = 0.121).

Three articles for gastric cancer (94, 102, 119) and 10 publications on prostate cancer (72, 77–79, 82, 83, 86, 87, 95, 110) with required data were included in the dose–response analyses. We found no significant associations between a 10-μg/dL increase in blood levels of lycopene and the risk of these two cancers (Table 3). In terms of breast cancer, 11 (66, 70, 74–76, 84, 97–99, 108, 118) and 12 papers (66, 70, 74–76, 84, 97–99, 108, 117, 118) had sufficient data for the non-linear and linear dose–response analyses, respectively. A non-significant inverse association was also observed for a 10-μg/dL elevate in lycopene levels and risk of breast cancer (Pooled RR: 0.98, 95% CI: 0.96–1.01, *p* = 0.225, Table 3). Regarding the non-linear dose–response analysis, no evidence of nonlinearity was observed for gastric, prostate, and breast cancers (P for nonlinearity > 0.10; Figures 5B–D).

#### Cancer mortality

The association between lycopene levels and overall cancer mortality was examined in 4 articles (104, 113, 114, 116), which enrolled 19,178 participants and 887 cases. We found an inverse significant association for cancer death, comparing the highest with the lowest concentration of lycopene (Pooled RR: 0.76, 95% CI: 0.60–0.98, I^2^ = 70.9%, *p* = 0.031). In terms of lung cancer mortality, such a significant association was also observed (Pooled RR: 0.65, 95% CI: 0.45–0.94, I^2^ = 0%, *p* = 0.022; Table 3). Data for other types of cancers were not sufficient for a meta-analysis. Also, we had insufficient data to perform the dose–response analyses.

### Sensitivity analyses, publication bias, and subgroup analyses

In the sensitivity analyses based on a fixed-effects model, the summary RRs obtained in the current meta-analysis were not driven by single studies. Based on Begg’s linear regression test, we found publication bias for the association between blood levels of lycopene and overall cancer risk (0.022) and between a 10-μg/dL increase in lycopene levels and overall (*p* < 0.001) and prostate cancer risk (*p* = 0.032). However, the application of the trim-and-fill method did not alter the pooled RRs, indicating that the results were not affected by the publication bias.

In the subgroup analyses, we found that the observed heterogeneity was explained by study location, sample size, adjustment for BMI, the tools used for dietary assessment and cancer diagnosis, follow-up duration, and quality of studies. Subgroup analyses for the association between total tomato intake and overall cancer risk, comparing the highest with the lowest tomato intake, revealed a significant inverse association between total tomato intake and cancer risk in studies that did not adjust for BMI (Table 1). For the association between dietary intake of lycopene and total cancer risk, significant interactions were found in terms of follow-up durations, adjustments for BMI, and methods used for cancer assessment (Table 2). A significant inverse association was found between lycopene intake and overall cancer risk in studies that were performed in non-US countries and those with high quality. In terms of blood levels of lycopene and total cancer risk, when comparing the highest with the lowest levels of lycopene, we found a significant inverse association between high-quality studies and those that were conducted in the US (Table 3).

### Overall findings based on a random-effects model

When we performed all analyses based on a random-effects model, our findings on cancer incidence remained unchanged (Supplementary Table 6). However, all significant inverse associations obtained for total tomato/lycopene intakes and blood levels of lycopene with cancer mortality became non-significant.

## Discussion

In this systematic review and meta-analysis, we found that higher levels of dietary and blood lycopene were, respectively, associated with 5 and 11% lower risk of overall cancer. In the dose–response analysis, each 10-μg/dL increase in blood levels of lycopene was associated with a 5% lower risk of overall cancer. Moreover, higher lycopene intakes/levels were negatively associated with lung, breast, and prostate cancers. Also, the association between lycopene intake and lung and prostate cancers was dose-dependent. For cancer mortality, higher total tomato/lycopene intakes and higher levels of blood lycopene were associated with a lower risk of overall cancer mortality. In the case of dietary lycopene, this association was dose-dependent based on the dose–response analyses.

The potential health benefits of tomatoes on cancer risk have been investigated in previous studies. However, the evidence seems to be conflicting (11, 44, 45, 49). In the present meta-analysis of prospective studies, no significant association was observed between total/raw tomato intake and risk of overall cancer and also breast and prostate cancers based on comparing the highest with the lowest intakes of total/raw tomato. In agreement with our findings, in a meta-analysis by Luo et al. (175), total tomato consumption was not associated with the risk of prostate cancer. Moreover, in two previous meta-analyses (138, 140), no significant association was observed between raw tomato intake and prostate cancer risk. However, Xu et al. (137) and Rowles et al. (138) indicated that total tomato intake was inversely associated with prostate cancer risk. It should be noted that Xu et al. (137) and Rowles et al. (138) combined effect sizes from case–control studies with those obtained from prospective studies. This difference might explain the disparity in the previous findings. In terms of breast cancer, a recent meta-analysis indicated no significant relationship with total tomato intake (144).

In contrast with tomato, we found that dietary lycopene intake was inversely associated with the risk of overall cancer and also lung cancer. It seems that an interaction between lycopene and other constituents in tomatoes results in a non-significant association between tomato intake and cancer risk. In addition, different cooking or processing methods may affect the properties of tomatoes. Recent studies have shown that cooked tomato has higher antioxidants compared to raw tomato. These antioxidants, such as FruHis, may help lycopene for its anticancer properties. Therefore, different findings on tomato and lycopene intake may be explained by the effects of processing methods on tomato properties.

Although we found no significant association between dietary intake of lycopene and the risk of prostate cancer, there was evidence of a linear link in this regard. However, by comparing the highest vs. the lowest levels of circulating lycopene, a negative association was observed. In line with our findings, three prior meta-analyses (139, 176, 177) indicated a significant inverse relationship between circulating lycopene and prostate cancer risk. However, Wang et al. (176) found a non-linear association between lycopene intake and risk of prostate cancer. Of the reasons explaining the discrepancy, one could be missing some eligible papers in the previous meta-analysis (37, 53). Also, the effect sizes from various observational studies (i.e., case–control, cross-sectional, cohort) were combined in the previous meta-analysis. In contrast to the meta-analyses mentioned above, a previous meta-analysis in 2013 (140) found no significant association between circulating levels of lycopene and prostate cancer risk. This difference is due to the lack of two eligible studies that were not included in the 2013 meta-analysis (88, 92). Additionally, the results of the present investigation did not support the inverse association between dietary and blood levels of lycopene and other types of cancers, including breast, ovarian, colorectal, gastric, and pancreatic cancer. These findings were in line with previous meta-analyses (141–143, 145, 178).

Lycopene intake in a usual diet is negligible, and it is difficult to investigate its association with health outcomes. Among the studies included in this meta-analysis, lycopene intake varied between 0.1 and 146.3 mg/day, which helped us to examine the relationship between lycopene intake and cancer risk at different levels of intake. However, due to the low dosage of lycopene in a diet, its estimation through dietary questionnaires is challenging. Thus, we evaluated blood levels of lycopene, which are the best indicators of lycopene intake. In most associations evaluated in the current meta-analysis, our findings regarding dietary lycopene intake were in line with those obtained for its blood levels. However, we found some differences in the risk of breast and prostate cancers that were inversely associated with blood lycopene levels but not dietary levels. These differences might be due to the low power of dietary questionnaires to estimate accurate dietary intakes. In addition, we found a significant inverse association for lung cancer risk in relation to dietary lycopene but not blood lycopene. Therefore, our findings on lung cancer should be considered with caution. Further studies are needed in this regard.

In the current study, total tomato intake was associated with a reduced risk of cancer mortality. However, this association with cancer incidence was not significant. This difference might be due to the duration of follow-up required for occurring outcomes. For survival studies, a short follow-up duration might be adequate for the incidence of cancer death. However, in prospective studies on healthy individuals, a long follow-up period is required for cancer incidence. Therefore, follow-up duration in the included studies on cancer incidence might be insufficient for cancer incidence. Future studies should consider this issue.

Some potential mechanisms could explain the cancer-protective effects of lycopene. Lycopene, as an antioxidant, exerts anticancer properties by inhibiting the production of insulin-like growth factor 1 and angiogenesis, promoting apoptosis and differentiation, and also protecting DNA and macromolecules from oxidation and carcinogens (179). Recent reports suggest that lycopene can suppress the proliferation of prostate cancer cells through the activation of peroxisome proliferator-activated receptor *γ* (PPARγ), liver X receptor *α* (LXRα), and ATP-binding cassette transporter ABCA1 (180). Additionally, lycopene could alleviate the prostate cancer risk by modulating the growth genes like cyclin-dependent protein kinase 7 (CDK7), B-cell lymphoma 2 (BCL2), epidermal growth factor receptor (EGFR), and insulin-like growth factor 1 (IGF-1) receptor (181).

In the present meta-analysis, we identified significant publication bias regarding the associations between blood levels of lycopene and the risk of overall cancer as well as prostate cancer. However, when we applied the trim-and-fill method, this publication bias was mitigated. This technique involved estimating and incorporating findings from potentially missing studies into the meta-analysis. By doing so, we created a hypothetical symmetry and assessed the overall effect size under conditions free from publication bias. Ultimately, this approach demonstrated that our results were not influenced by publication bias.

### Strengths and weaknesses of this study

The present meta-analysis has some strengths. First, including prospective studies with a large number of participants and cancer cases allowed us to quantitatively investigate the association between tomato/lycopene intake and blood levels of lycopene with cancer risk and mortality. Second, linear and non-linear dose–response analyses were performed to reach compelling evidence for the quantitative evaluation of relationships. Third, due to the prospective design of included studies, the effect of selection and recall bias is negligible.

However, our findings should be interpreted by considering some limitations. Although the included studies had controlled their analyses for potential confounders, the role of residual or unmeasured confounders, like dietary intakes of other food groups or nutrients, cannot be ruled out. Additionally, in some included studies, the confounding effects of important variables such as energy intake and BMI were not taken into account. Moreover, a number of studies in this review did not have sufficient data to be included in the dose–response meta-analyses. Also, because of the limited number of studies, we were not able to assess the relationship between exposures to other types of cancers like endometrial, hepatocellular, renal, head and neck, and skin cancers. Different approaches that were used for the assessment of exposures and outcomes among included studies are other limitations of this meta-analysis. However, subgroup analysis was performed to control for these differences. Lastly, most studies evaluated tomato and lycopene intakes based on a single measurement at the baseline of the study, and dietary changes during the follow-up were not considered.

## Conclusion

In conclusion, lycopene (both dietary intake and blood levels) was inversely linked with overall cancer risk. There was also evidence of a linear relationship in the case of lycopene levels so that each 10 μg/dL increase in blood levels of lycopene was associated with a 5% lower risk of overall cancer. In terms of specific cancers, we found a linear inverse association between lycopene consumption and prostate cancer risk and a significant inverse association between blood lycopene levels and the risk of breast and prostate cancers. Regarding cancer mortality, total tomato/lycopene intakes and blood levels of lycopene were associated with a lower risk of cancer mortality. Also, the association between dietary lycopene and cancer mortality was non-linear, so the highest risk reduction was observed in the dosages between 5 and 8 mg/day. As most of the included studies were conducted in Western countries, the generalizability of findings to the worldwide population should be done with caution. Thus, further studies are warranted to affirm our findings.

## Data Availability

The original contributions presented in the study are included in the article/Supplementary material, further inquiries can be directed to the corresponding author.
