# Supplementary material for: Dietary intake of tomato and lycopene, blood levels of lycopene, and risk of total and specific cancers in adults: a systematic review and dose–response meta-analysis of prospective cohort studies
Source: Front Nutr. 2025 Feb 12;12:1516048. doi: 10.3389/fnut.2025.1516048 (PMC11860085; doi:10.3389/fnut.2025.1516048)
Supplement: Supplementary file 1 [file Table_1.docx]

**Supplementary Table 1**: The terms used to search relevant publications on the relation between tomato and lycopene with risk of cancer and its mortality

| **In PubMed, Scopus, and ISI Web of Science** | | **n** |
| --- | --- | --- |
|  | 1. Lycopene |  |
|  | 2. “Lycopersicon esculentum” |  |
|  | 3. carotenoids |  |
|  | 4. antioxidants |  |
|  | 5. Tomato |  |
|  | 6. “solanum lycopersicum” |  |
|  | 7. “tomato product” |  |
|  | 8. (1 OR 2 OR 3 OR 4 OR 5 OR 6 OR 7) |  |
|  | 9. Neoplasms |  |
|  | 10. Cancer |  |
|  | 11. Carcinoma |  |
|  | 12. Death |  |
|  | 13. “Fatal outcome” |  |
|  | 14. Survival |  |
|  | 15. (9 OR 10 OR 11 OR 12 OR 13 OR 14) |  |
|  | 16. Prospective |  |
|  | 17. Cohort |  |
|  | 18. Longitudinal |  |
|  | 19. Follow-up |  |
|  | 20. Nested |  |
|  | 21. Hazard |  |
|  | 22. (16 OR 17 OR 18 OR 19 OR 20 OR 20 OR 21) |  |
|  | (8 AND 15 AND 22) **In PubMed** | 390 |
|  | (1 OR 5 AND 10 AND 10 OR 16 OR 17 OR 18 OR 19 OR 20 OR 21) **In Scopus** | 1093 |
|  | (1 AND 10) **In ISI Web of Science** | 195 |
|  | **Duplicate** | 302 |
| **In Google Scholar** | | 600 |
|  | Tomato and cancer  Lycopene and cancer  By searching the above combinations in this engine, we screened the first 300 relevancy ranked papers to avoid missing any eligible studies. |  |
| **Total by considering duplicated papers** | | 2580 |

**Supplemental Table 2**: Summary of prospective cohort studies on the meta-analysis of tomato and lycopene intake with risk of cancer and its mortality

| Author | Country, study name | Age, y^1^ | Sample size, *n* | Follow-up, y^2^ | Cases, *n* | Exposure | Exposure assessment | Outcome | Outcome assessment | Median/cutoff point | RR (95% CI) | Adjustment |
| --- | --- | --- | --- | --- | --- | --- | --- | --- | --- | --- | --- | --- |
| Ambrosini et al.  2008 | Australia, FWRWG | ≥18 | M: 11798 | 14 | 97 | Raw tomato | FFQ, self-reported | Prostate cancer | Medical records | 0-1.7 serv/wk  >1.7-4.1 serv/wk  >4.1 serv/wk | 1  1.04 (0.62-1.73)  1.04 (0.60-1.80) | Age, total fruit and vegetable intake (except  b-carotene and retinol models), randomly assigned retinol or b-carotene supplement, and source of crocidolite exposure |
| Cui et al. 2008 | US, WHI | 50-79 | F: 84805 | 7.6 | 2879 | Dietary lycopene | FFQ, self-reported | Breast cancer | Self-reported | <4.22 mg  4.22-5.97 mg  5.97-7.89 mg  7.89-10.82 mg  ≥10.82 mg | 1  0.98 (0.87-1.10)  1.09 (0.97-1.22)  0.95 (0.84-1.08)  0.93 (0.82-1.06) | Energy intake, age at baseline, ethnicity, educational level, age at menarche, age at menopause, parity, age at first full-term pregnancy, oral contraceptive use, postmenopausal hormone use, BMI, physical activity, alcohol drinking, dietary folate intake, tobacco smoking, hysterectomy, bilateral oophorectomy, history of benign breast disease, and family history of breast cancer |
| Cui et al. 2011 | US, NHS | 30-55 | F: 121701 | 26 | 669 | Dietary lycopene | FFQ, self-reported | Endometrial cancer | Self-reported | 1.84 mg/d  3.44 mg/d  4.41 mg/d  5.53 mg/d  7.44 mg/d | 1  1.01 (0.79-1.28)  0.93 (0.73-1.19)  0.90 (0.70-1.15)  1.09 (0.86-1.37) | Energy intake, smoking, oral contraceptive use, postmenopausal hormone use, age at menopause, hypertension, diabetes, and BMI |
| Fraser et al. 2020 | US, AHS-2 | 30-104 | M: 27934 | 7.9 | 1226 | Raw tomato | FFQ, self-reported | Prostate cancer | Self-reported | Never/rarely  1-3 times/mo  1 time/wk  2-4 times/wk  5-6 times/wk  Daily | 1  1.06 (0.81-1.38)  0.80 (0.61-1.05)  0.97 (0.76-1.24)  0.86 (0.65-1.13)  1.02 (0.77-1.36) | Ethnicity, family history of prostate cancer, educational level, never smoking, ever been diagnosed with BPH, years since last PSA screening, dairy consumption, energy intake, and being vegan |
|  |  |  |  |  |  | Dietary lycopene |  |  |  | >10.22 vs. <1.89 mg/d | 0.94 (0.80-1.12) |  |
| Giovannucci et al. 1995 | US, HPFS | 40-75 | M: 47894 | 6 | 764 | Tomatoes | FFQ, self-reported | Prostate cancer | Self-reported | 0  1-3 serv/mo  1 serv/wk  2-4 serv/wk | 1  0.90 (0.72-1.13)  0.91 (0.75-1.11)  0.74 (0.58-0.93) | Energy intake, age |
| Han et al. 2013 | US, VITAL | 50-76 | M/F: 77446 | 8 | 764 | Dietary lycopene | FFQ, self-reported | Pancreatic Cancer | Medical records | 0-3.77 mg  3.78-7.22 mg  7.23-218.76 mg | 1  0.79 (0.54-1.17)  0.82 (0.53-1.26) | Age, gender, ethnicity, education, BMI, physical activity, cigarette smoking status, total alcohol consumption, family history of pancreatic cancer, history of diabetes, and energy intake |
| Ho et al. 2015 | US, WHI | 50-79 | F: 196196 | 20 | 240 | Dietary lycopene | FFQ, self-reported | Renal cell cancer | Self-reported | <2.73 mg  2.73-4.24 mg  4.25-6.43 mg  ≥6.44 mg | 1  0.98 (0.68-1.42)  0.74 (0.50-1.11)  0.61 (0.39-0.97) | All of the micronutrients, age, clinical trial, ethnicity, education, BMI, hypertension, smoking status, oral contraceptive use, hysterectomy ever, oophorectomy ever, physical activity, and energy intake. |
| Holick et al. 2002 | Finland, ATBC | 50-69 | M: 27084 | 14 | 1644 | Dietary lycopene | DHQ, self-reported | Lung cancer | Medical records | <0.23 mg  0.23-0.47 mg  0.48-0.73 mg  0.74-1.17 mg  >1.17 mg | 1  0.93 (0.81-1.07)  0.93 (0.81-1.08)  0.79 (0.68-0.92)  0.72 (0.61-0.84) | Age, years smoked, cigarettes per day, intervention (α-tocopherol and β-carotene supplement), supplement use (β-carotene and vitamin A), energy intake, cholesterol, and fat |
| Holick et al. 2005 | US, NHS | 30-55 | F: 121700 | 20 | 237 | Dietary lycopene | FFQ, self-reported | Bladder cancer | Self-reported | 0.84 mg/d  1.76 mg/d  4.10 mg/d  6.42 mg/d  11.18 mg/d | 1  1.03 (0.66-1.60)  1.57 (1.04-2.37)  0.84 (0.53-1.33)  1.18 (0.78-1.79) | Age, pack-years of cigarette smoking, current smoking, and energy intake |
| Kim et al. 2019 | US, NHS and HPFS | 40-75 | M: 48400 | 26 | 2222 | Dietary lycopene | FFQ, self-reported | Squamous Cell Carcinoma | Self-reported | 3.24 mg/d  5.08 mg/d  6.65 mg/d  8.70 mg/d  12.88 mg/d | 1  0.97 (0.84-1.11)  0.89 (0.77-1.03)  0.84 (0.72-0.98)  0.87 (0.75-1.01) | Age, family history of melanoma, natural hair color, number of arm moles, sunburn susceptibility as a child or adolescent (no experience, no reaction or some redness, burn, or painful burn or blisters), number of lifetimes blistering sunburns, cumulative UV flux since baseline, BMI, physical activity, smoking status, personal history of basal cell carcinoma, melanoma, or nonskin cancer, energy intake, alcohol intake, and caffeine intake, menopausal status, and postmenopausal hormone use |
|  |  | 30-55 | F: 75170 | 28 | 1756 |  |  |  |  | 3.31 mg/d  4.70 mg/d  5.85 mg/d  7.29 mg/d  10.13 mg/d | 1  1.03 (0.91-1.17)  0.96 (0.84-1.09)  0.89 (0.78-1.02)  0.87 (0.76-0.99) |  |
| Kirsh et al. 2006 | US, PLCO | 55-74 | M: 29361 | 8 | 1338 | Dietary lycopene | FFQ, self-reported | Prostate cancer | Medical records | 5.05 mg/d  7.55 mg/d  9.65 mg/d  12.27 mg/d  17.59 mg/d | 1  1.10 (0.93-1.30)  1.06 (0.89-1.25)  1.07 (0.90-1.27)  0.95 (0.79-1.13) | Age, energy intake, ethnicity, study center, family history of prostate cancer, BMI, smoking status, physical activity, supplemental vitamin E intake, total fat intake, red meat intake, history of diabetes, aspirin use, and previous number of screening exams within the follow-up period |
| Larsson et al. 2007 | Sweden, SMC and CSM | 45-83 | M/F: 82002 | 8 | 139 | Dietary lycopene | FFQ, self-reported | Gastric cancer | Medical records | 0.74 mg/d  1.50 mg/d  2.26 mg/d  3.54 mg/d | 1  0.97 (0.62-1.52)  1.15 (0.72-1.82)  0.92 (0.53-1.58) | Age, gender, education, diabetes, smoking status and pack-years of smoking, and energy intake |
| Larsson et al. 2010 | Sweden, SMC | ≥18 | F: 36664 | 9 | 1008 | Dietary lycopene | FFQ, self-recorded | Breast cancer | Medical records | <1.06 mg/d  1.06-1.66 mg/d  1.67-2.23 mg/d  2.24-2.97 mg/d  ≥2.98 mg/d | 1  1.01 (0.82-1.24)  1.03 (0.84-1.27)  1.15 (0.94-1.41)  0.91 (0.73-1.14) | Age, education, family history of breast cancer, history of benign breast disease, parity, age at first birth, age at menarche, age at menopause, use of oral contraceptives, use of postmenopausal hormones, BMI, physical activity, smoking, multivitamin use, energy intake, and alcohol intake |
| Malila. et al. 2002 | Finland, ATBC | 50-69 | M: 26951 | 8 | 184 | Dietary lycopene | Questionnaire, self-reported | Colorectal cancer | Medical records | 0.15 mg/d  0.43 mg/d  0.77 mg/d  1.46 mg/d | 1  1.32 (0.87-2.00)  1.32 (0.87-2.02)  1.06 (0.68-1.66) | Age, BMI, alcohol intake, serum cholesterol, physical activity, cigarettes/day, and trial supplementation |
| Michaud et al. 1999 | US, HPFS | 40-75 | M: 47909 | 10 | 252 | Dietary lycopene | FFQ, self-reported | Bladder cancer | Self-reported | 3.41 mg/d  6.16 mg/d  8.68 mg/d  12.20 mg/d  18.89 mg/d | 1  1.28 (0.85-1.91)  1.40 (0.94-2.07)  1.22 (0.81-1.82)  0.98 (0.65-1.49) | Age, pack-years of cigarette smoking, current smoking status, geographic region, total fluid intake, and energy intake |
| Michaud et al. 2000 | US, NHS and HPFS | 40-75 | M: 46924 | 10 | 275 | Dietary lycopene | FFQ, self-reported | Lung cancer | Self-reported | >15.09 vs. <5.01 mg/d | 0.86 (0.59-1.25) | Age, smoking status, age at start of smoking, energy intake, and time period |
|  |  | 30-55 | F: 77283 | 12 | 519 |  |  |  | Medical records | >12.59 vs. <5.42 mg/d | 0.77 (0.60-1.00) |  |
| Michaud et al. 2002 | Finland, ATBC | 50-69 | M: 27111 | 13 | 344 | Dietary lycopene | Questionnaire, self-reported | Bladder cancer | Medical records | 0.12 mg/d  0.35 mg/d  0.59 mg/d  0.91 mg/d  1.59 mg/d | 1  0.97 (0.69-1.37)  1.07 (0.76-1.50)  1.08 (0.77-1.52)  1.32 (0.95-1.83) | Age, duration of smoking, smoking dose, energy intake, and trial intervention |
| Navarro Silvera et al. 2006 | Canada, NBSS | 40-59 | F: 48776 | 20 | 264 | Dietary lycopene | FFQ, self-reported | Ovarian cancer | Medical records | 0-4.71 mg/d  >4.71-8.60 mg/d  >8.60-15.00 mg/d  >15.00 mg/d | 1  0.88 (0.61-1.27)  0.95 (0.66-1.37)  0.92 (0.63-1.34) | Age, pack-years of smoking, menopausal status, use of oral contraceptives, BMI, participation in vigorous physical activity, energy intake at baseline, study center, and randomization group |
| Neuhouser et al. 2003 | US, CARET | 45-60 | M/F: 7048 | 12 | 326 | Dietary lycopene | FFQ, self-reported | Lung cancer | Medical records | ≤2.48 mg/day  2.49-3.68 mg/day  3.69-4.98 mg/day  4.99-7.07 mg/day  ≥7.08 mg/day | 1  0.86 (0.61-1.21)  0.88 (0.63-1.23)  0.89 (0.64-1.25)  0.94 (0.67-1.32) | Gender, age, smoking status, total pack-years of smoking, asbestos exposure, ethnicity, and enrollment center |
| Nouraie et al. 2005 | Finland, ATBC | 50-69 | M: 27110 | 14 | 57 | Dietary lycopene | FFQ, self-reported | Gastric cardia  Cancer | Medical records | ≤31 mg/d  0.32-0.62 mg/d  0.63-1.07 mg/d  ≥1.08 mg/d | 1  1.29 (0.62-2.68)  1.06 (0.48-2.33)  0.97 (0.41-2.34) | Age, total years of smoking, education, and dietary nitrate, BMI, educational level, family history of breast cancer, smoking status, and alcohol intake, use of multivitamin supplement, hormone use, reproductive history and dietary fat, and fiber intake |
|  |  |  |  |  | 163 |  |  | Gastric non-cardia cancer |  | ≤31 mg/d  0.32-0.62 mg/d  0.63-1.07 mg/d  ≥1.08 mg/d | 1  0.58 (0.37-0.90)  0.80 (0.53-1.23)  0.62 (0.37-1.03) |  |
| Pantavos et al. 2014 | Netherland, RS | ≥55 | F: 3209 | 21 | 199 | Dietary lycopene | FFQ, self-reported | Breast cancer | Medical records | 10.29 mg  69.39 mg  146.33 mg | 1  1.06 (0.74-1.49)  1.03 (0.73-1.45) | Age, BMI, educational level, family history of breast cancer, smoking status, alcohol intake, use of multivitamin supplement, hormone use, and reproductive history and dietary fat and |
| Park et al. 2009 | US, MEC | 45-75 | M: 85898 | 9 | 1165 | Dietary lycopene | FFQ, self-reported | Colorectal cancer | Medical records | <0.75 mg/1000kcal  0.75-<1.07 mg/1000kcal  1.07-<1.43 mg/1000kcal  1.43-<2.02 mg/1000kcal  ≥2.02 mg/1000kcal | 1  1.00 (0.83-1.19)  1.18 (0.99-1.41)  1.12 (0.93-1.35)  1.16 (0.96-1.41) | Age at cohort entry, family history of colorectal cancer, history of intestinal polyps, pack-years of cigarette smoking, BMI, hours of vigorous activity, use of nonsteroidal anti-inflammatory drugs, multivitamin use, energy intake, alcohol intake, red meat intake, dietary fiber intake, total calcium intake (foods and supplements), total vitamin D intake (foods and supplements), total folate intake (foods and supplements), and use of hormone replacement therapy (for women only) |
|  |  |  | F: 105106 |  | 920 |  |  |  |  | < 0.80 mg/1000kcal  0.80-<1.14 mg/1000kcal  1.14-<1.54 mg/1000kcal  1.54-<2.23 mg/1000kcal  ≥2.23 mg/1000kcal | 1  0.99 (0.81-1.21)  0.91 (0.74-1.11)  0.94 (0.76-1.15)  1.01 (0.82-1.25) |  |
| Park et al. 2015 | US, MEC | 45-75 | M: 75216 | 17 | 7115 | Dietary lycopene | FFQ, self-reported | Prostate cancer | Medical records | <0.75 mg/1000kcal  0.75-<1.07 mg/1000kcal  1.07-1.43 mg/1000kcal  1.43-2.02 mg/1000kcal  ≥2.02 mg/1000kcal | 1  1.12 (0.94-1.34)  1.10 (0.92-1.31)  1.06 (0.89-1.27)  1.10 (0.93-1.31) | Age at cohort entry, ethnicity, family history of prostate cancer, and the other risk factors |
| Schuurman et al. 2002 | Netherland, NLCS | 55-69 | M: 58279 | 6.3 | 642 | Dietary lycopene | FFQ, self-reported | Prostate cancer | Medical records | 0.10 mg/d  0.40 mg/d  0.70 mg/d  1.10 mg/d  2.00 mg/d | 1  0.79 (0.57-1.09)  1.08 (0.80-1.47)  0.99 (0.72-1.36)  0.98 (0.71-1.34) | Age, family history of prostate cancer, socioeconomic status, and alcohol from white or fortified wine |
| Stram et al. 2006 | US, MEC | 45-75 | M: 82486 | 8 | 3922 | Tomatoes | FFQ, self-reported | Prostate cancer | Medical records | ≤12.00 g/1000kcal  >12.00-≤18.20 g/1000kcal  >18.20-≤25.40 g/1000kcal  >25.40-≤37.30 g/1000kcal  >37.30 g/1000kcal | 1  1.06 (0.96-1.18)  1.08 (0.98-1.20)  1.02 (0.92-1.14)  1.02 (0.92-1.14) | Age, BMI, education, and family history of prostate cancer |
| Thomson et al. 2008 | US, WHI | 50-79 | F: 133614 | 8.3 | 352 | Dietary lycopene | FFQ, self-reported | Ovarian cancer | Self-reported | <2.74 mg  2.74-<4.21 mg  4.21-<6.32 mg  >6.32 mg | 1  1.03 (0.75-1.40)  1.24 (0.92-1.69)  1.02 (0.73-1.43) | Age, log calories, no breast/ovary cancer relatives, dietary modification randomization arm, hysterectomy status, minority ethnicity, pack-years smoking, physical activity, nonsteroidal anti-inflammatory drug use, parity, infertility, duration of oral contraceptive use, lifetime ovulatory cycles, partial oophorectomy, age at menopause, and HT usage at entry |
| Xu et al. 2021 | US, PLCO | 55-74 | M/F: 101683 | 12.5 | 774 | Dietary lycopene | DHQ, self-reported | Bladder cancer | Medical records | ≤2.79 mg/d  ≥2.80-≤4.06 mg/d  ≥4.07-≤5.61 mg/d  ≥5.62-≤8.44 mg/d  ≥8.45 mg/d | 1  1.02 (0.81-1.27)  0.88 (0.70-1.12)  0.95 (0.75-1.21)  1.04 (0.82-1.33) | Age, gender, ethnicity, BMI, education, smoking status, drinking status, energy intake, randomization arm, family history of any cancer, and marital status |
|  |  |  |  |  |  | Raw tomato |  |  |  | ≤3.57 g/d  ≥3.60-≤9.41 g/d  ≥9.44-≤17.56 g/d  ≥17.67-≤32.44 g/d  ≥32.64 g/d | 1  1.17 (0.93-1.46)  1.14 (0.91-1.43)  1.19 (0.94-1.50)  1.20 (0.95-1.52) |  |
| Xu et al. 2021 | US, PLCO | 55-74 | M/F: 101832 | 22 | 7161 | Dietary  lycopene | DHQ, self-reported | Cancer mortality | Medical records | <2.79 mg/d  ≥2.79-<4.06 mg/d  ≥4.06-<5.61 mg/d  ≥5.61-<8.44 mg/d  ≥8.44 mg/d | 1  0.91 (0.85-0.98)  0.95 (0.88-1.03)  0.89 (0.82-0.96)  0.95 (0.87-1.04) | Age, gender, ethnicity, BMI, education, smoking status, marital status, randomization arm, aspirin use, history of hypertension, history of diabetes, history of stroke, history of heart attack, vegetables intake, fruit intake, alcohol intake, and energy intake |
|  |  |  |  |  |  | Raw tomato |  |  |  | ≤3.63 g/d  ≥3.65-≤9.53 g/d  ≥9.55-≤17.56 g/d  ≥17.67-≤32.44 g/d  ≥32.64 g/d | 1  1.02 (0.95-1.10)  1.01 (0.93-1.09)  1.03 (0.96-1.11)  1.04 (0.96-1.13) |  |
| Yuan et al. 2003 | China, SCHC | 45-74 | M/F: 62392 | 7 | 482 | Dietary lycopene | FFQ, interview | Lung cancer | Medical records | 0.07 mg/1000kcal  0.24 mg/1000kcal  0.45 mg/1000kcal  0.75 mg/1000kcal  1.49 mg/1000kcal | 1  1.11 (0.88-1.43)  0.77 (0.58-1.02)  0.89 (0.67-1.18)  0.89 (0.65-1.21) | Age at baseline, gender, dialect group, year of interview, level of education, BMI, cigarettes smoked per day, number of years of smoking, and number of years since quitting smoking for former smokers |
| Zhang et al. 1999 | US, NHS | 33-60 | F:  83234 | 14 | 784 | Dietary lycopene | FFQ, self-reported | Premenopausal breast cancer | Self-reported | 10.55-13.85 vs. 0.53-2.80 mg/d | 1.10 (0.87-1.38) | Age, length of follow-up, total energy intake, parity, age at first birth, age at menarche, history of breast cancer in mother or a sister, history of benign breast, alcohol intake, BMI, height in inches, variables for age at menopause and for postmenopausal hormone use |
|  |  |  |  |  | 1913 |  |  | Postmenopausal breast cancer |  | 10.55-13.85 vs. 0.53-2.80 mg/d | 1.02 (0.88-1.18) |  |
| Zu et al. 2014 | US, HPFS | 40-75 | M:  49898 | 24 | 5728 | Dietary lycopene | FFQ, self-reported | Prostate cancer | Self-reported | 0-3.68 mg/d  3.69-5.30 mg/d  5.31-7.06 mg/d  7.07-10.13 mg/d  10.14-115.01 mg/d | 1  1.00 (0.95-1.10)  0.96 (0.89-1.00)  0.96 (0.88-1.00)  0.91 (0.84-1.00) | Age, height, dietary intakes of calcium, α-linolenic acid, coffee, energy intake |
| Mazidi et al. 2020 | US, NHANES | ≥20 | M/F:  22835 | 11 | 796 | Tomato | Recall, interview | Cancer mortality | Medical records | >1.05 vs. <0.16 cups/d | 0.88 (0.84-0.93) | Age, gender, ethnicity, education, marital status, poverty to income ratio, total energy intake, physical activity, smoking, alcohol consumption, BMI, dietary fat, C-reactive protein, carbohydrates, dietary fiber, meat, BMI, hypertension and diabetes |
|  |  |  |  |  |  | Dietary lycopene |  |  |  | 5.93-12.46 vs. 0.26-0.80 mg/d | 0.82 (0.8-0.85) |  |
| Wang et al. 2016 | US, CPS II | ≥18 | M:  8898 | 20 | 526 | Dietary lycopene | FFQ, self-reported | Prostate cancer mortality | Medical records | <2.8 mg/d  2.82- <4.2 mg/d  4.22- <6.1 mg/d  6.1 - 30.2 mg/d | 1  0.89 (0.71-1.13)  0.85 (0.67-1.09)  1.00 (0.85-1.15) | Age at diagnosis, ethnicity, calendar year of diagnosis, tumor extent, nodal involvement, Gleason score, history of pre diagnosis prostate specific antigen testing, education, physical activity, BMI, and smoking |
|  |  |  |  |  | 526 | Tomato |  |  |  | <2.20 serv/wk  2.22- <3.60 serv/wk  3.62- <5.80 serv/wk  5.82- <45.10 serv/wk | 1  1.03 (0.80-1.31)  1.06 (0.82-1.33)  1.02 (0.87-1.18) |  |
| Diallo et al. 2016 | France, SU.VI.MAX | 51.8 | M:  3313 | 13 | 139 | Tomato products | 24-h dietary record | Prostate cancer | Self-reported | <172.50 g/d  172.50-193.80 g/d  >193.80 g/d | 1  1.12 (0.72-1.71)  1.43 (0.94-2.16) | Age, energy intake without alcohol, intervention group of the initial SU.VI.MAX trial, number of 24-h dietary records, smoking status, educational level, physical activity, height, BMI, alcohol intake, family history of prostate cancer, baseline plasma prostate-specific antigen concentration, Ca intake, dairy product intake and plasma α-tocopherol and Se concentrations |
| Dunneram et al. 2019 | UK, UKWCS | 35-69 | F:  29183 | 21 | 1625 | Tomato | FFQ, self-reported | Breast cancer | Medical records | Per 83 g/d increment | 0.86 (0.69-1.08) | Age, ethanol intake, duration of breastfeeding, physical activity, smoking, social class, menopausal status, history of diabetes, and history of hypertension |
|  |  |  | 9227 |  | 86 |  |  | Endometrial cancer |  | Per 83 g/d increment | 0.63 (0.3-1.34) |  |
|  |  |  | 29229 |  | 251 |  |  | Ovarian Cancer |  | Per 83 g/d increment | 1.05 (0.65-1.68) |  |
| Kiani et al. 2006 | US, AHS | ≥25 | F:  13281 | 16 | 53 | Tomato | FFQ, self-reported | Epithelial ovarian cancer | Medical records | 0-<1 serv/wk  ≥1 serv/wk-<5 serv/wk  ≥5 serv/wk | 1  0.71 (0.34-1.48)  0.33 (0.13-0.82) | Age, parity and BMI, and also for age at menopause and hormone replacement therapy in postmenopausal analyses |
| Lin et al. 2006 | US, NHS and HPFS | 30-55 | F:  71976 | 10 | 498 | Tomato | FFQ, self-reported | Colorectal Cancer | Self-reported | 0–2 serv/wk  3–4 serv/wk  5–6 serv/wk  1 serv/d  ≥2 serv/d | 1  1.18(0.92-1.52)  1.22 (0.92-1.61)  1.06 (0.71-1.59)  1.02 (0.70-1.48) | Age, BMI, family history of colorectal cancer, history of colorectal polyps, prior sigmoidoscopy screening, physical activity, smoking status, red meat intake, alcohol consumption, total energy intake, total calcium intake, total folate intake, total fiber intake, aspirin use, and multivitamin use, postmenopausal hormone replacement therapy |
|  |  | 40-75 | M:  35425 |  | 380 |  |  |  |  | 0–2 serv/wk  3–4 serv/wk  5–6 serv/wk  1 serv/d  ≥2 serv/d | 1  1.10 (0.83-1.46)  1.11 (0.80-1.54)  1.10 (0.70-1.73)  1.23 (0.84-1.80) |  |
| Masala et al.  2012 | Italy, EPIC | 36-64 | F:  32578 | 15 | 1072 | Tomato | FFQ, self-reported | Breast  cancer | Medical records | <29.70 g/d  29.70–47.90 g/d  47.90–68.80 g/d  68.80–101.40 g/d  >101.40 g/d | 1  0.91 (0.75-1.10)  1.01 (0.83-1.22)  1.06 (0.87-1.28)  0.83 (0.68-1.02) | weight, height, education school, number of children, age at menarche, menopausal status, energy intake except alcohol, alcohol intake, current use of hormone therapy, smoking status, and physical activity |
| Mills et al.  1989 | US, AHS | ≥25 | M:  14000 | 6 | 180 | Tomato | Recall, interview | Prostate cancer | Medical records | <1 serv/wk  l-4 serv/wk  ≥5 serv/wk | 1  0.64 (0.42-0.97)  0.60 (0.37-0.97) | Age; education; current use of meat, poultry, or fish; current fish only; beans; legumes or peas; citrus fruit; dry fruit; index of fruit, nuts, and tomatoes |
| Nothlings et al.  2006 | US, HLMCS | 45-75 | M/F:  183522 | 9 | 529 | Tomato | FFQ, self-reported | Exocrine pancreatic cancer | Medical records | 8.33 g/1000 kcal/d  15.25 g/1000 kcal/d  22.04 g/1000 kcal/d  31.26 g/1000 kcal/d  53.01 g/1000 kcal/d | 1  0.84 (0.65-1.09)  0.90 (0.69-1.18)  0.94 (0.72-1.23)  0.83 (0.62-1.10) | Gender and time in the study, age at cohort entry, ethnicity, history of diabetes mellitus, family history of pancreatic cancer, smoking status, pack-years of smoking, intakes of red meat and processed meat, energy intake, and BMI |
| Perez-Cornago et al.  2017 | European countries, EPIC | 35-70 | M:  142239 | 21 | 7036 | Tomato | Dietary questionnaires & recall, self-reported | Prostate cancer | Self-reported | ≤9.00 g/d  >9.00-≤18.90 g/d  >18.90-≤ 30.80 g/d  >30.80-≤ 67.30 g/d  >67.30 g/d | 1  1.04 (0.97-1.12)  1.03 (0.95-1.11)  1.00 (0.92-1.08)  1.08 (0.97-1.19) | Age, stratified by recruitment center and age at recruitment, educational level, smoking status, marital status, diabetes, physical activity, height |
| Suzuki et al.  2013 | Japan, JPHC | 57.2 | F:  47289 | 12 | 452 | Tomato | FFQ, self-reported | Breast  cancer | Medical records | 1.7 g/d  7.4 g/d  22.7 g/d  66.7 g/d | 1  0.84 (0.64-1.10)  0.86 (0.66-1.13)  0.99 (0.76-1.29) | Age, area, height, recent BMI, BMI at age 20 years, age at menarche, age at first birth, parity, menopausal status, use of exogenous female hormones, smoking status, leisure-time physical activity, alcohol intake, total energy-adjusted intake of isoflavones, and vitamin C supplement, fruits |
| Takachi et al.  2010 | Japan, JPHC | 45-74 | M:  43475 | 9 | 339 | Tomato and tomato products | FFQ, self-reported | Prostate cancer | Medical records | 1.2 g/d  6 g/d  25 g/d  68 g/d | 1  1.06 (0.77-1.46)  1.08 (0.79-1.49)  1.16 (0.84-1.58) | Age, public health center area, BMI, smoking status, alcohol consumption, quartile of dairy food, quartile of soy products, green tea consumption, vitamin supplement use, marital status, screening examination |
| Colditz et al.  1985 | US,  MHCPS | ≥65 | M/F:  1226 | 4.75 | 317 | Tomato | FFQ, self-reported | Cancer mortality | Medical records | ≥1 time/ wk vs. <1time/wk | 0.50 (0.30-0.80) | Age |
| Sakauchi et al.  2004 | Japan,  JACC | ≥40 | M/F:  114517 | 11 | 63 | Tomato | FFQ, self-reported | Urothelial Cancer Death | Medical records | ≤1–2 serv/mo  1–2 serv/wk  ≥3–4 serv/wk | 1  0.96 (0.53-1.75)  0.78 (0.42-1.44) | Gender, age, and Smoking Index |
| Takata et al.  2013 | China,  SMHS | 40-74 | M:  61092 | 7 | 359 | Tomato | FFQ/ interview | Lung cancer | Self-reported | 3.5 g/d  13.9 g/d  28.9 g/d  69.3 g/d | 1  0.85 (0.65-1.12)  0.79 (0.58-1.08)  0.99 (0.73-1.34) | Age, year of smoking, the number of cigarettes smoked per day, current smoking status, total caloric intake, education, BMI, ever consumption of tea, history of chronic bronchitis, family history of lung cancer among first-degree relatives, total fruit intake for each vegetable, and total vegetable intake for each fruit |
| Kristal et al.  2010 | US,  PCPT | 63.1 | M:  9559 | 9 | 1576 | Dietary lycopene | FFQ, self-reported | Prostate cancer  (GS 2–7) | Medical records | <3.99 mg/d  3.99–6.65 mg/d  6.65–10.92 mg/d  >10.92 mg/d | 1  1.11 (0.97-1.25)  1.00 (0.87-1.15)  1.05 (0.90-1.21) | Age, ethnicity, family history of prostate cancer in first-degree relatives, treatment arm, and BMI |
|  |  |  |  |  | 127 |  |  | Prostate cancer  (GS 8–10) |  | <3.99 mg/d  3.99–6.65 mg/d  6.65–10.92 mg/d  >10.92 mg/d | 1  1.22 (0.73-2.02)  1.49 (0.90-2.47)  1.32 (0.76-2.31) |  |
| Agalliu et al.  2011 | Canada,  CSDLH | 67.7 | M:  2525 | 11 | 661 | Dietary lycopene | FFQ, self-reported | Prostate cancer | Medical records | 2.45 mg/d  4.87 mg/d  6.77 mg/d  9.61 mg/d  15.87 mg/d | 1  0.71 (0.53-0.96)  0.77 (0.58-1.03)  0.77 (0.57-1.03)  0.82 (0.61-1.10) | Age at baseline, ethnicity, BMI, exercise activity, and education |
| Botterweck et al.  2000 | Netherland,  NLCS | 55- 69 | M/F:  3405 | 6 | 282 | Dietary lycopene | FFQ, self-reported | Gastric cancer | Medical records | 0.15 mg/d  0.48 mg/d  0.81 mg/d  1.21 mg/d  2.20 mg/d | 1  0.70 (0.50-1.10)  0.90 (0.60-1.40)  1.10 (0.70-1.50)  1.00 (0.70-1.50) | Age, gender, smoking status, education, stomach disorders, and family history of stomach carcinoma |
| de Munter et al.  2015 | Netherlan, NLCS | 55-69 | M/F:  4313 | 20 | 415 | Dietary lycopene | FFQ, self-reported | Overall head and neck cancer | Medical records | 0.19 mg/d  0.61 mg/d  1.03 mg/d  1.90 mg/d | 1  0.91 (0.66-1.25)  0.87 (0.61-1.23)  0.98 (0.65-1.45) | Age, gender, cigarette smoking, alcohol intake, and total daily energy intake |
| Jain et al.  2000 | Canada,  NBSS | 40-59 | F:  3918 | 13 | 221 | Dietary lycopene | FFQ, self-reported | Endometrial cancer | Medical records | <4.44 mg/d  4..44-8.07 mg/d  8.08-13.95 mg/d  >13.95 mg/d | 1  0.80 (0.54-1.17)  0.81 (0.56-1.18)  0.63 (0.43-0.94) | Total energy, age, BMI, ever smoker, used oral contraceptives, used hormone replacement therapy, university education, live births, age at menarche |
| Lane et al.  2016 | UK,  UDCC | 50-69 | M:  5245 | 13.3 | 1717 | Dietary lycopene | Record | Prostate cancer | Medical records | <0.35 mg/d  0.35-0.77 mg/d  0.78-1.30 mg/d  1.31-2.14 mg/d  >2.14 mg/d | 1  1.10 (0.88-1.38)  1.17 (0.94-1.47)  1.02 (0.81-1.28)  0.85 (0.67-1.07) | Age, BMI, socioeconomic, smoking and marital status, diabetes and energy intake |
| Rohan et al.  2002 | Canada,  NBSS | 40-59 | F:  5516 | 13 | 155 | Dietary lycopene | FFQ, self-reported | Lung cancer | Medical records | <4.46 mg/d  4.46- 8.13 mg/d  8.14-14.11 mg/d  >14.11 mg/d | 1  0.86 (0.52-1.43)  1.18 (0.72-1.96)  1.04 (0.61-1.76) | Age, study allocation, study center, cigarette smoking, vitamin C intake, folate intake, dietary fiber intake, and energy intake |
| Steinmetz et al.  1993 | US, IWHS | 55-69 | F:  2952 | 4 | 138 | Dietary lycopene | FFQ, self-reported | Lung Cancer | Medical records | Q4 vs. Q1 | 1.21 (0.69-2.10) | Age. energy intake, pack-years of smoking in multivariate logistic regression |
|  |  |  |  |  |  | Tomato |  |  |  | ≤0.5 serv/wk  1 serv/wk  ≥3 serv/wk | 1  1.57 (1.00-2.50)  1.00 (0.61-1.64) |  |
| Terry et al.  2002 | Canada,  NBSS | 40-59 | F:  5629 | 13 | 295 | Dietary lycopene | FFQ, self-reported | Colorectal Cancer | Medical records | ≤3.92 mg/d  >3.92–6.88 mg/d  >6.88–10.44 mg/d  >10.44–16.87 mg/d  >16.87 mg/d | 1  1.10 (0.70-1.60)  0.80 (0.50-1.30)  0.70 (0.50-1.10)  1.00 (0.70-1.60) | Age, smoking, BMI, hours of vigorous physical activity, education, multivitamin use, and intakes of total energy, total fat, alcohol, dietary fiber, folate, calcium |
| Terry et al.  2002 | Canada,  NBSS | 40-59 | F:  6691 | 13 | 1452 | Dietary lycopene | FFQ/ self-reported | Breast  cancer | Medical records | 2.28 mg/d  5.34 mg/d  8.49 mg/d  13.04 mg/d  23.75 mg/d | 1  1.18 (0.90-1.46)  0.94 (0.70-1.17)  0.96 (0.70-1.19)  1.14 (0.90-1.41) | Age, screening center, allocation, smoking, BMI, hours of vigorous physical activity, education, family history of breast cancer, history of benign breast disease, age at menarche, parity, menopausal status, oral contraceptive use, hormone replacement therapy, practiced breast self-examination, multivitamin use, intakes of total energy, alcohol, dietary fiber, folate, calcium |
| Thomas et al.  2020 | Singapore,  SCHS | 45-74 | M/F:  61321 | 22 | 561 | Dietary lycopene | FFQ, interview | Hepatocellular carcinoma | Medical records | <0.26 mg/d  0.26-0.69 mg/d  0.69-1.36 mg/d  ≥1.36 mg/d | 1  0.90 (0.71-1.14)  0.93 (0.73-1.17)  0.91 (0.71-1.18) | Age, gender, dialect, BMI, education, smoking status, alcohol intake, year of enrollment, diabetes status, physical activity, and total energy intake. |
|  |  |  |  |  |  | Tomato |  |  |  | <1.95 g/d  1.95-4.81 g/d  4.81-8.83 g/d  ≥8.83 g/d | 1  0.70 (0.56-0.88)  0.73 (0.58-0.92)  0.63 (0.49-0.81) |  |
| Voorrips et al.  2000 | Netherlands,  NLCS | 55-69 | M:  2464 | 6 | 939 | Dietary lycopene | FFQ, self-reported | Lung cancer | Medical records | 0.13 mg/d  0.25 mg/d  0.72 mg/d  1.09 mg/d  2.03 mg/d | 1  0.87 (0.63-1.19)  1.09 (0.79-1.49)  1.18 (0.85-1.62)  1.05 (0.75-1.46) | Current smoking, years of smoking cigarettes, number of cigarettes per day, highest educational level, family history of lung cancer, age |
| Zeegers et al.  2001 | Netherlands,  NLCS | 55-69 | M/F:  3692 | 6 | 569 | Dietary lycopene | FFQ, self-reported | Bladder cancer | Medical records | 0.15 mg/d  0.48 mg/d  0.81 mg/d  1.21 mg/d  2.21 mg/d | 1  0.85 (0.61-1.18)  1.07 (0.77-1.48)  1.18 (0.84-1.65)  1.08 (0.77-1.51) | Age, gender, cigarette smoking amount, cigarette smoking duration |
| Sesso et al.  2005 | US,  WHS | 53.9 | F:  1016 | 9.9 | 508 | Dietary lycopene | FFQ, interview | Breast  cancer | Medical records | 3.33 mg/d  5.43 mg/d  7.68 mg/d  10.81 mg/d  16.74 mg/d | 1  0.95 (0.77-1.18)  1.00 (0.80-1.24)  1.10 (0.89-1.36)  1.00 (0.80-1.25) | Age, smoking status, randomized aspirin treatment, randomized vitamin E treatment, randomized h-carotene treatment, plasma cholesterol level, BMI, family history of breast cancer, physical activity, age at menarche, ever use of oral contraceptives, age at first pregnancy, number of pregnancies, postmenopausal status, postmenopausal hormone use, alcohol intake, fiber intake, folate intake, saturated fat intake, and fruit and vegetable intake |
|  |  |  |  |  |  | Tomato |  |  |  | 0  1-3 serv/mo  1-4 serv/wk  ≥5 serv/wk | 1  1.54 (1.04-2.29)  1.54 (1.05-2.24)  1.45 (0.94-2.22) |  |
| Schuurman et al.  1998 | Netherlands,  NLCS | 55-69 | M:  1066 | 6 | 610 | Tomato | FFQ, interview | Prostate cancer | Medical records | Q4 vs. Q1 | 1.10 (0.80-1.51) | Age, family history of prostate cancer, socioeconomic status, total fruit and total vegetable consumption. |
| Maasland et al.  2014 | Netherlands,  NLCS | 55-69 | M/F:  4313 | 20 | 415 | Tomato | FFQ, self-reported | Head and neck cancer | Medical records | Per 25 g/d increment | 1.04 (0.89-1.21) | Age, gender, cigarette smoking, alcohol consumption, total vegetable and fruit intake |
| Boggs et al.  2010 | US, BWHS | 21-69 | F:  51928 | 12 | 1268 | Tomato | FFQ, self-reported | Breast  cancer | Self-reported | <1 serv/mo  1–3 serv/mo  1–2 serv/wk  ≥3 serv/wk | 1  0.92 (0.80-1.06)  0.93 (0.79-1.10)  1.04 (0.85-1.27) | Age, energy intake, age at menarche, BMI at age 18 years, family history of breast cancer, education, geographic region, parity, age at first birth, oral contraceptive use, menopausal status, age at menopause, menopausal hormone use, vigorous activity, smoking status, alcohol intake, and multivitamin use. |
| Heinen et al.  2012 | Netherlands,  NLCS | 55-69 | M/F:  4291 | 16 | 423 | Dietary lycopene | FFQ, self-reported | Pancreatic Cancer | Medical records | 0.15 mg/d  0.50 mg/d  0.80 mg/d  1.20 mg/d  2.15 mg/d | 1  0.91 (0.65-1.27)  0.85 (0.61-1.20)  1.10 (0.80-1.52)  1.03 (0.74-1.43) | Age, gender, smoking, BMI, family history of pancreatic cancer, history of diabetes mellitus, intake of energy, red meat, coffee, alcohol |
| Steevens et al.  2011 | Netherlands,  NLCS | 55-70 | M/F:  4136 | 16 | 101 | Tomato | FFQ, self-reported | Esophageal squamous cell carcinoma | Medical records | Per 25 g/d increment | 0.87 (0.62-1.22) | Age, gender, cigarette smoking, alcohol consumption, consumption of red meat, consumption of fish |
|  |  |  |  |  |  |  |  |  |  |  |  |  |
|  |  |  | 4191 |  | 156 |  |  | Gastric cardia adenocarcinoma |  | Per 25 g/d increment | 0.93 (0.71-1.23) |  |
|  |  |  |  |  |  |  |  |  |  |  |  |  |
|  |  |  | 4495 |  | 460 |  |  | Gastric noncardia adenocarcinoma |  | Per 25 g/d increment | 1.13 (1.00-1.28) |  |
| Stolzenberg-Solomon et al.  2002 | Finland,  ATBC | 50-69 | M:  27111 | 12 | 163 | Dietary lycopene | DHQ, self-reported | Pancreatic Cancer | Medical records | 0.12 mg/d  0.35 mg/d  0.62 mg/d  0.94 mg/d  1.28 mg/d | 1  0.92 (0.57-1.48)  0.93 (0.57-1.51)  1.01 (0.62-1.65)  1.06 (0.64-1.77) | Energy intake by the residual method, age ,years of smoking, energy-adjusted folate intake |
| Koushik et al.  2006 | US and Europe,  CPS II, NC, IWHS, NLCS, NYSC, NHS, NHS II, SMC, and WHS | ≥18 | F:  521911 | 7-22 | 2012 | Dietary lycopene | FFQ, self-reported | Epithelial ovarian cancer | Self-reported | Q5 vs. Q1 | 0.97 (0.84-1.12) | Parity, oral contraceptive use, menopausal status and postmenopausal hormone use, age at menarche, BMI, physical activity, smoking status, total energy intake, age |
| Cho et al.  2003 | US,  NHS II | 25-42 | F:  90655 | 8 | 714 | Dietary lycopene | FFQ, self-reported | Breast cancer | Self-reported | 3.57 mg/d  5.53 mg/d  7.64 mg/d  10.49 mg/d  15.74 mg/d | 1  1.01 (0.79-1.30)  0.91 (0.71-1.18)  1.19 (0.94-1.50)  1.17 (0.92-1.49) | Smoking, height, parity and age at first birth, BMI, age at menarche, family history of breast cancer, history of benign breast disease, oral contraceptive use, menopausal status, alcohol intake, energy, animal fat |
| Farvid et al.  2018 | US,  NHS | 30-55 | F:  88301 | 32 | 7599 | Tomato | FFQ, self-reported | Invasive  breast cancer | Self-reported | ≤2 serv/wk  >2- 4 serv/wk  >4- 6 serv/wk  >6 serv/wk- 1 serv/d  >1 serv/d | 1  1.02 (0.97-1.07)  1.03 (0.97-1.09)  1.02 (0.93-1.11)  1.06 (0.98-1.15) | Family history of breast cancer, history of benign breast disease, height, BMI, weight change since age 18 years, physical activity, oral contraceptive use, alcohol intake, total energy intake, age at menarche, parity and age at first birth, menopausal status, age at menopause, past postmenopausal hormone use |
|  | NHS II | 25-42 | F:  93844 | 22 | 3300 |  |  |  |  |  |  |  |
| Horn-Ross et al.  2002 | US,  CTS | 21-103 | F:  111526 | 2 | 711 | Dietary lycopene | FFQ, self-reported | Invasive  breast cancer | Medical records | 0.60 mg/d  1.22 mg/d  1.08 mg/d  2.46 mg/d  3.09 mg/d | 1  1.00 (0.80-1.30)  1.00 (0.80-1.20)  1.00 (0.80-1.30)  0.90 (0.70-1.10) | Age, ethnicity, daily caloric intake, family history of breast cancer, age at menarche, nulliparity/age at first full-term pregnancy, physical activity, BMI, menopausal status |

Abbreviation: BMI, body mass index; CI, confidence interval; RR, risk ratio; Q, quartile; M, male; mo, month; n, number; y, year; wk, week; US, the United States; kcal, kilocalories; EPIC, European Prospective Investigation into Cancer and Nutrition; FFQ, food frequency questionnaire; DHQ, dietary history questionnaire; F, female; g/d, gram(s) per day; mg/d, milligram(s) per day; HPFS, Health Professional Follow-up Study; IWHS, Iowa Women’s Health Study; NLCS, Netherlands Cohort Study; NHS, Nurse Health Study; NHS II, Nurse Health Study II; Q, quartile or quintile; serv, serving; SMHS, Shanghai Men’s Health Study; FWRWG, Former Workers and Residents of Wittenoom Gorge; WHI, Women’s Health Initiative Study; AHS, Adventist Health Study; AHS II, Adventist Health Study II; VITAL, The Vitamins and Lifestyle Study; ATBC, Alpha-Tocopherol, Beta-Carotene Cancer Prevention Study; PLCO, The Prostate, Lung, Colorectal, and Ovarian Trial, SMC, The Swedish Mammography Cohort; CSM, Cohort of Swedish Men; NBSS, Canadian National Breast Screening Study; RS, The Rotterdam study; MEC, The Multiethnic Cohort Study; SCHS, The Singapore Chinese Health Study; NHANES, National Health and Nutrition Examination Survey; CPS II, Cancer Prevention Study II; SU.VI.MAX, Supplémentation en Vitamines et Minéraux Antioxydants; UKWCS, UK Women’s Cohort Study; HLMCS, Hawaii–Los Angeles Multiethnic Cohort Study; JPHC, Japan Public Health Center-based Prospective Study; JACC, Japan Collaborative Cohort Study study; PCPT, Prostate Cancer Prevention Trial; CSDLH, Canadian Study of Diet, Lifestyle and Health; BWHS, Black Women’s Health Study; CTS, The California Teachers Study; UDCCS, UK Dietary Cohort Consortium studies; CARET, B-Carotene and Retinol Efficacy Trial; NYSC; New York State Cohort, NC; Nutrition Cohort

^1^Presented as mean or range

^2^ Presented as max or mean

**Supplemental Table 3**: Summary of prospective cohort studies on the meta-analysis of blood levels of lycopene with risk of cancer and its mortality

| Author | Country, study name | Age, y^1^ | Sample size, *n* | Follow-up, y^2^ | Cases, *n* | Exposure | Exposure assessment | Outcome | Outcome assessment | Median/cutoff point | RR (95% CI) | Adjustment |
| --- | --- | --- | --- | --- | --- | --- | --- | --- | --- | --- | --- | --- |
| Bakker et al.  2016 | Europe,  EPIC | 49.99 | F:  3004 | 12 | 515 | Plasma lycopene | HPLC | ER - breast cancer | Medical records | 6.09 µg/dL  14.00 µg/dL  20.13 µg/dL  27.39 µg/dL  39.84 µg/dL | 1  0.71 (0.50-1.02)  0.83 (0.54-1.27)  0.59 (0.36-0.95)  1.07 (0.56-2.03) | Age, menopausal status at recruitment, use of exogenous hormones, phase of menstrual cycle, fasting status at blood collection, time of blood collection, BMI, height, age at menarche, age at first full-term pregnancy, oral contraceptive use, hormone therapy use, smoking status, alcohol consumption, educational level, intake of saturated fatty acids, energy intake, and season of blood collection |
|  |  |  |  |  | 636 |  |  | ER + breast cancer |  | 5.85 µg/dL  13.91 µg/dL  19.71 µg/dL  27.39 µg/dL  40.20 µg/dL | 1  0.81 (0.53-1.25)  0.94 (0.61-1.44)  1.37 (0.88-2.11)  0.90 (0.55-1.48) |  |
| Batieha et al.  1993 | US,  NR | ≥18 | F:  150 | 6-7.5 | 50 | Serum lycopene | HPLC | Cervical cancer | Medical records | <24.90 µg/dL  24.90-41.80 µg/dL  >41.80 µg/dL | 1  0.96 (0.95-0.98)  0.40 (0.15-1.04) | Education, contraceptive use, marital status, smoking habits, retinol, lutein, alpha-tocopherol, gama-tocopherol, and selenium |
| Beilby et al.  2010 | Australia,  ABA | 69.55 | F:  321 | 14 | 96 | Serum lycopene | HPLC | Prostate cancer | Medical records | 0-0.19 µmol/L  0.20-0.30 µmol/L  0.31-1.30 µmol/L | 1  0.55 (0.30-0.99)  0.77 (0.40-1.47) | Age, and vitamin A supplement |
| Breslow et al.  1995 | US,  NR | ≥18 | M/F:  90 | ≥ 18 | 30 | Serum lycopene | HPLC | Melanoma cancer | Medical records | High vs. low | 1.10 (0.40-3.20) | Smoking, education, hours since the last meal did not substantially change the results |
|  |  |  | 96 |  | 32 |  |  | Basal cell skin cancer |  | High vs. low | 1.40 (0.40-4.00) |  |
|  |  |  | 111 |  | 37 |  |  | Squamous cell skin cancer |  | High vs. low | 1.00 (0.30-3.10) |  |
| Dorgan et al.  1998 | US, CMBC | 61.6 | F:  308 | 12 | 105 | Serum lycopene | HPLC | Breast cancer | Medical records | ≤0.22 µmol/L  0.23-0.31 µmol/L  0.32-0.50 µmol/lL  0.51-1.75 µmol/L | 1  1.20 (0.60-2.40)  1.10 (0.50-2.20)  0.50 (0.20-1.20) | Total serum cholesterol concentration, packs of cigarettes smoked/day, BMI |
| Dorjgochoo et al.  2009 | China,  SWHS | 40-70 | F:  1091 | 10 | 365 | Plasma lycopene | HPLC | Breast cancer | Medical records | 4.83 µg/dL  8.92 µg/dL  14.61 µg/dL  21.89 µg/dL | 1  1.03 (0.68-1.57)  1.26 (0.83-1.94)  0.83 (0.49-1.39) | Age at entry, education, occupation, age at menarche, age at 1st live birth, waist to hips ratio , exercised regularly in past 5 years, ever smoke , menopausal status, history of breast fibroadenoma , 1st degree family history breast cancer , total intakes of energy: vegetables, fruit, red meat and fish, regular tea consumption, other plasma lipophilic antioxidants |
| Eliassen et al.  2015 | US,  NHS | 30-55 | F:  4293 | 20 | 2147 | Plasma lycopene | HPLC | Breast cancer | Medical records | <288 µg/dL  288 to <371 µg/dL  371 to <449 µg/dL  449 to <563 µg/dL  ≥563 µg/dL | 1  1.03 (0.85-1.25)  0.80 (0.66-0.98)  0.87 (0.71-1.06)  0.82 (0.67-1.01) | BMI at age 18 y, weight gain since age 18 y, ages at menarche, first birth, and menopause, parity, alcohol intake, history of benign breast disease, family history of breast cancer |
| Epplein et al.  2009 | US,  MEC | 45-75 | F:  821 | 2.5 | 286 | Plasma lycopene | HPLC | Breast cancer | Medical records | ≤218.7 ng/mL  218.8-292.2 ng/mL  292.3-391.7 ng/mL  ≥391.8 ng/mL | 1  1.04 (0.68-1.58)  0.91 (0.06-1.37)  0.88 (0.57-1.38) | Age at blood draw and fasting hours prior to blood draw, BMI, alcohol use, age at menarche, age at menopause, age at first birth, and number of full-term pregnancies |
| Gann et al.  1999 | US,  PHS | 40-84 | M:  1872 | 13 | 578 | Plasma lycopene | HPLC | Prostate cancer | Medical records | 21.57 µg/dL  30.76 µg/dL  39.82 µg/dL  51.15 µg/dL  64.87 µg/dL | 1  0.89 (0.64-1.23)  0.90 (0.65-1.24)  0.87 (0.63-1.19)  0.75 (0.54-1.06) | Exercise frequency, BMI, plasma total cholesterol, alcohol, and multivitamin supplement use |
| Gill et al.  2009 | US,  MC | 45-75 | M:  1403 | 2.5 | 467 | Serum lycopene | HPLC | Prostate cancer | Medical records | 22.00 µg/dL  33.90 µg/dL  46.20 µg/dL  65.60 µg/dL | 1  0.96 (0.67-1.38)  0.85 (0.59-1.22)  0.78 (0.53-1.14) | Age at specimen collection and fasting hours prior to blood draw as continuous variables, BMI, family history of prostate cancer, education |
| Goodman et al.  2003 | US,  CARET | 45-69 | M/F:  579 | 14 | 277 | Serum lycopene | HPLC | Lung cancer | Medical records | 16 µg/dL  26.6 µg/dL  37.8 µg/dL  49.6 µg/dL | 1  1.04 (0.64-1.69)  0.71 (0.43-1.17)  0.86 (0.52-1.43) | Study center at randomization, age at randomization within 5-year intervals, gender, smoking status at randomization, and year of randomization, and controlling for pack-years of smoking and years quit smoking |
|  |  |  | M:  616 |  | 307 |  |  | Prostate cancer |  | 18.3 µg/dL  27.5 µg/dL  36.9 µg/dL  46.5 µg/dL | 1  0.65 (0.36-1.15)  0.47 (0.26-0.87)  1.04 (0.61-1.77) |  |
| Helzlsouer et al.  1989 | US,  NR | 11-98 | M/F:  105 | 12 | 35 | Serum lycopene | HPLC | Bladder Cancer | Medical records | 27 µg/dL  43 µg/dL  59 µg/dL | 1  0.48 (0.47-0.49)  0.49 (0.15-1.59) | Cigarette smoking, vitamin supplements |
| Helzlsouer et al.  1996 | US,  NR | 45-54 | F:  102 | 16 | 35 | Serum lycopene | HPLC | Ovarian Cancer | Medical records | <21.90 µg/dL  21.90-35.20 µg/dL  >35.20 µg/dL | 1  1.14 (0.40-3.20)  1.36 (0.40-4.30) | None |
| Hsing et al.  1990 | US,  NR | ≥18 | M:  206 | 13 | 103 | Serum lycopene | HPLC | Prostate cancer | Medical records | <20 µg/dL  20-32 µg/dL  32-47 µg/dL  >47 µg/dL | 1  0.81 (0.38-1.73)  0.55 (0.23-1.34)  0.50 (0.20-1.29) | Effects of cigarette smoking, hours since last meal, years of education |
| Huang et al.  2003 | US,  CLUE I | 45-64 | M:  546 | 22 | 182 | Serum lycopene | HPLC | Prostate cancer | Medical records | <21.7 µg/dL  21.7-31.1 µg/dL  31.1-41.1 µg/dL  41.1-54.9 µg/dL  >54.9 µg/dL | 1  0.86 (0.51-1.47)  0.74 (0.41-1.33)  0.96 (0.55-1.67)  0.83 (0.46-1.48) | Total lipid levels in the blood, hours since last meal, education, BMI |
|  | US,  CLUE II |  | 426 | 7 | 142 |  |  |  |  | < 24.3 µg/dL  24.3-35.2 µg/dL  35.2-48.8 µg/dL  48.8-62.8 µg/dL  >62.8 µg/dL | 1  0.88 (0.45-1.70)  0.77 (0.40-1.47)  0.83 (0.42-1.62)  0.79 (0.41-1.54) |  |
| Hulten et al.  2001 | Sweden,  VIP, MONICA, and MSP | ≥40 | M/F:  624 | 12 | 201 | Plasma lycopene | HPLC | Breast cancer | Medical records | 4.83 µg/dL  11.27 µg/dL  17.58 µg/dL  23.75 µg/dL | 1  1.10 (0.60-1.80)  0.80 (0.40-1.30)  1.00 (0.60-1.80) | BMI, plasma total cholesterol and triglycerides |
| Jeurnink et al.  2015 | Europe,  EPIC | 57.9 | M/F:  892 | 5.25 | 446 | Plasma lycopene | HPLC | Pancreatic cancer | Medical records and self-reported | <163.14 nmol/L  163.51-258.59 nmol/L  258.99-394.82 nmol/L >396.50 nmol/L | 1  0.96 (0.62-1.47)  1.08 (0.71-1.66)  1.00 (0.62-1.62) | Smoking status, duration and intensity of smoking, cotinine levels, waist circumference and diabetes status |
| Key et al.  2007 | Europe,  EPIC | 60.25 | M:  2030 | 4 | 966 | Plasma lycopene | HPLC | Prostate cancer | Medical records | <15.04 µg/dL  15.04-<24.32 µg/dL  24.32-<34.75 µg/dL  34.75-<49.37 µg/dL  ≥49.37 µg/dL | 1  1.36 (1.02-1.83)  1.25 (0.93-1.68)  1.11 (0.83-­1.49)  0.97 (0.70­­­-1.34) | BMI, smoking status, alcohol intake, physical activity level, marital status, and educational level. |
| Kristal et al.  2011 | US,  PCPT | ≥55 | M:  2908 | 7 | 1157 | Serum lycopene | HPLC | Prostate cancer (Gleason 2–6) | Medical records | <26.30 µg/dL  26.30- <36.00 µg/dL  36.00 -<46.60 µg/dL  ≥46.60 µg/dL | 1  0.82 (0.66-1.02)  0.93 (0.74-1.16)  0.91 (0.72-1.14) | Age, ethnicity, diabetes, serum cholesterol, BMI |
|  |  |  | 2212 |  | 461 |  |  | Prostate cancer (Gleason 7-10) |  | <26.30 µg/dL  26.30- <36.00 µg/dL  36.00 -<46.60 µg/dL  ≥46.60 µg/dL | 1  1.19 (0.89-1.60)  0.93 (0.68-1.28)  1.16 (0.85-1.58) |  |
|  |  |  | 1876 |  | 125 |  |  | Prostate cancer (Gleason 8-10) |  | <26.30 µg/dL  26.30- <36.00 µg/dL  36.00 -<46.60 µg/dL  ≥46.60 µg/dL | 1  0.99 (0.59-1.66)  0.87 (0.50-1.51)  1.20 (0.71-2.04) |  |
| Lee et al.  2021 | US,  SCCS | 40-79 | M:  983 | 4.4 | 343 | Plasma lycopene | HPLC | Prostate cancer | Medical records | T3 vs. T1 | 0.51 (0.29-0.87) | Education, family income, history of diabetes, history of prostate cancer, NPB score, comorbidity index, BMI, smoking, alcohol consumption, physical activity, Healthy Eating Index, blood retinol and carotenoids, and time from meal to blood collection |
| Leenders et al.  2014 | Europe,  EPIC | 35-70 | M/F:  1796 | 8 | 898 | Plasma lycopene | HPLC | Colon cancer | Medical records | 5.10 µg/dL  11.20 µg/dL  19.45 µg/dL  29.85 µg/dL | 1  0.99 (0.74-1.33)  1.31 (0.97-1.77)  1.11 (0.80-1.54) | Matching factors and physical activity, smoking status, number of cigarettes smoked per day, smoking duration, time since stopped smoking, alcohol consumption at baseline, waist circumference and highest level of education. |
|  |  |  | 1002 | 501 |  |  |  | Rectal cancer |  | 5.10 µg/dL  11.20 µg/dL  19.45 µg/dL  29.85 µg/dL | 1  0.93 (0.63-1.37)  1.36 (0.92-2.01)  1.21 (0.77-1.87) |  |
| Nomura et al.  1997 | US,  NR | 52-74 | M:  284 | 4 | 142 | Serum lycopene | HPLC | Prostate cancer | Medical records | Q4 vs. Q1 | 1.10 (0.50-2.20) | None |
| Nomura et al.  2003 | US,  NR | 73.2 | M:  219 | 4 | 109 | Serum lycopene | HPLC | Bladder Cancer | Medical records | 7.83 µg/dL  12.15 µg/dL  18.39 µg/dL  26.56 µg/dL | 1  0.65 (0.31-1.38)  0.77 (0.37-1.62)  0.60 (0.28-1.29) | Pack-years of cigarette smoking, age |
| Nordstrom et al.  2016 | US,  NR | 59 | M:  559 | 7 | 112 | Plasma lycopene | HPLC | High-Grade Prostate Cancer | Medical records | Q4 vs. Q1 | 0.55 (0.28-1.08) | Age at diagnosis, circulating cholesterol, smoking status at diagnosis, Caucasian ethnicity |
| Peng et al.  2020 | US,  NHS | 30-55 | F:  3614 | 19 | 1919 | Plasma lycopene | HPLC | Breast cancer | Self-reported | Q4 vs. Q1 | 0.85 (0.70-1.03) | Age at blood draw, BMI at blood draw, family history, history of benign breast disease, alcohol consumption, age at first birth/parity, menopausal status, age at menarche, smoking status |
|  | NHS II | 25-42 |  | 12 |  |  |  |  |  |  |  |  |
| Persson et al.  2008 | Japan,  JPHC  (Cohort I) | 40-59 | M/F:  1022 | 14 | 511 | Plasma lycopene | HPLC | Gastric cancer | Medical records | 0 µg/dl  4.90 µg/dl  9.90 µg/dl  19.20 µg/dl | 1  1.45 (1.00–2.12)  0.93 (0.60–1.44)  1.02 (0.66–1.58) | Family history of gastric cancer, Helicobacter pylori status, smoking, BMI, salt intake and consumption of highly salted foods, plasma levels of cholesterol |
|  | (Cohort II) | 40-69 |  | 11 |  |  |  |  |  |  |  |  |
| Peters et al.  2007 | US,  PLCO | 55-74 | M:  1536 | 8 | 692 | Serum lycopene | HPLC | Prostate cancer | Self-reported | 30.5 µg/dL  46.8 µg/dL  62.2 µg/dL  78.5 µg/dL  108.4 µg/dL | 1  1.00 (0.72-1.40)  0.93 (0.66-1.31)  1.16 (0.84-1.61)  1.14 (0.82-1.58) | Age, time since initial screening, year of blood draw, study center |
| Ros et al.  2012 | Europe,  EPIC | 25-70 | M/F:  1712 | 13 | 856 | Plasma lycopene | HPLC | Urothelial cell carcinoma | Medical records | ≤240.61 nmol/L  240.84–369.26 nmol/L  369.97–531.15 nmol/L  ≥532.19 nmol/L | 1  1.00 (0.75-1.34)  0.69 (0.51-0.94)  0.96 (0.69-1.33) | Age at blood collection, study center, gender, date of blood collection, time of blood collection, fasting status, smoking status, duration, and intensity |
| Sato et al.  2002 | US,  CLUE I and II | 51.2 | F:  488 | 20 | 244 | Serum lycopene | HPLC | Breast cancer | Medical records | <20.10 µg/dL  20.10-28.70 µg/dL  28.80-37.60 µg/dL  37.70-49.20 µg/dL  ≥49.30 µg/dL | 1  0.78 (0.45-1.34)  0.53 (0.29-0.97)  0.48 (0.26-0.88)  0.55 (0.29-1.06) | Total serum cholesterol, smoking, BMI, age at first birth, family history of breast cancer, history of benign breast disease, and total cholesterol |
| Sesso et al.  2005 | US,  WHS | 53.9 | F:  1016 | 7 | 508 | Plasma lycopene | HPLC | Breast cancer | Medical records | 5.60 µg/dL  8.50 µg/dL  11.20 µg/dL  15.90 µg/dL | 1  0.95 (0.59-1.55)  1.15 (0.69-1.90)  0.93 (0.56-1.52) | Age, smoking status, randomized aspirin treatment, randomized vitamin E treatment, randomized h-carotene treatment, plasma cholesterol level, BMI, family history of breast cancer, physical activity, age at menarche, ever use of oral contraceptives, age at first pregnancy, number of pregnancies, postmenopausal status, postmenopausal hormone use, alcohol intake, fiber intake, folate intake, saturated fat intake, and fruit and vegetable intake |
| Sisti et al.  2015 | US,  NHS | 30-55 | F:  2358 | 21 | 1179 | Plasma lycopene | HPLC | Breast cancer | Self-reported | <33 µg/dL  ≥33-<43 µg/dL  ≥43-<55 µg/dL  ≥55 µg/dL | 1  0.81 (0.64-1.04)  0.92 (0.72-1.17)  0.80 (0.62-1.02) | BMI at blood draw, age at menarche, alcohol intake, parity/age at first birth), family history of breast cancer , history of benign breast disease |
|  | NHS II | 25-42 |  | 15 |  |  |  |  |  |  |  |  |
| Wang et al.  2015 | US,  CPS II | 69.4 | F:  992 | 9 | 496 | Plasma lycopene | HPLC | Breast cancer | Self-reported | <273.60 µg/dL  273.60–<366.20 µg/dL  366.20–<484.80 µg/dL  ≥484.80 µg/dL | 1  0.77 (0.50-1.16)  0.70 (0.45-1.09)  0.95 (0.60-1.50) | Matching factors, history of benign breast disease, combination of age of mother at first birth and number of live births, BMI, alcohol consumption, smoking status, other plasma carotenoids (except for total carotenoids) and total fruit and vegetable intake |
| Wu et al.  2004 | US,  HPFS | 40-75 | M:  900 | 5 | 450 | Plasma lycopene | HPLC | Prostate cancer | Medical records | Q5 vs. Q1 | 0.66 (0.38-1.13) | Matching variables, cholesterol levels, selenium supplementation, vitamin E supplementation, family history of prostate cancer, BMI, height, vigorous exercise, history of vasectomy and current smoking using |
| Yu et al.  1999 | Taiwan,  NR | 30-65 | M:  459 | 8 | 84 | Plasma lycopene | HPLC | Hepatocellular Carcinoma | Medical records | ≤12.10 µg/dL  ≤16.10 µg/dL  ≤24.50 µg/dL  >24.50 µg/dL | 1  1.65 (0.72-3.79)  1.61 (0.72-3.63)  1.40 (0.54-3.60) | Hepatitis B surface antigen (HBsAg) carrier status, age, plasma retinol levels, alcohol drinking, cigarette smoking |
| Yuan et al.  2004 | China,  SCS | 45-64 | M:  761 | 12 | 191 | Serum lycopene | HPLC | Gastric Cancer | Medical records | <1.20 µg/dL  1.20-2.50 µg/dL  2.60-4.60 µg/dL  >4.60 µg/dL | 1  0.89 (0.53-1.49)  0.80 (0.46-1.37)  0.63 (0.34-1.15) | Age, month and year of biospecimen collection, and neighborhood of residence at recruitment, cigarette smoking, alcohol consumption |
| Yuan et al.  2006 | China,  SCS | 45-65 | M:  1300 | 15 | 213 | Serum lycopene | HPLC | Hepatocellular Carcinoma | Medical records | Q4 vs. Q1 | 0.84 (0.42-1.68) | Cigarette smoking, heavy alcohol consumption, self-reported history of physician-diagnosed hepatitis or liver cirrhosis at recruitment, seropositivity for hepatitis B surface antigen, serum levels of β -carotene, α -tocopherol, and γ -tocopherol |
| Ito et al.  2005 | Japan,  JACC | 40-79 | M:  538 | 11 | 163 | Serum lycopene | HPLC | Lung cancer mortality | Medical records | <0.04 µmol/L  0.04-0.06 µmol/L  0.07-0.14 µmol/L  ≥0.15 µmol/L | 1  1.07 (0.58-1.95)  0.59 (0.30 -1.17)  0.44 (0.19 -1.05) | Age, participating institution, smoking, alcohol drinking, BMI, serum total cholesterol levels |
|  |  |  | F:  160 |  | 48 |  |  |  |  | <0.07 µmol/L  0.07-0.11 µmol/L  0.12-0.19 µmol/L  ≥0.20 µmol/L | 1  1.17 (0.35-3.96)  0.92 (0.25-3.42)  0.63 (0.12-3.25) |  |
| Ito et al.  2005 | Japan,  NR | 39-79 | M/F:  3182 | 10.5 | 132 | Serum lycopene | HPLC | All cancer mortality | Medical records | High vs. low | 0.61 (0.39-0.97) | Gender, age, smoking habit, and serum levels of total cholesterol and ALT activity |
|  |  |  |  |  | 31 |  |  | Lung cancer mortality |  | High vs. low | 0.93 (0.39-2.24) |  |
| Toniolo et al.  2001 | US,  WHS | 35-65 | F:  540 | 9 | 270 | Serum lycopene | HPLC | Breast cancer | Medical records | Q4 vs. Q1 | 0.67 (0.39-1.14) | Age at first full-term pregnancy, family history of breast cancer, history of benign breast disease, and total cholesterol |
| Jenab et al.  2006 | Europe,  EPIC | 59.1 | M/F:  889 | 3.5 | 244 | Plasma lycopene | HPLC | Gastric adenocarcinomas | Medical records | <17.80 µg/dL  ≥17. 80-<29.20 µg/dL  ≥29.20-<44.70 µg/dL  ≥44.80 µg/dL | 1  0.89 (0.56-1.40)  0.86 (0.55-1.36)  0.63 (0.36-1.09) | BMI, total energy intake, smoking status/duration/intensity and Hp status |
| Dorgan et al.  2004 | US,  ISO-BCC | 40-75 | M/F:  376 | 9.5 | 221 | Serum lycopene | HPLC | Basal cell carcinoma | Self-reported | <30.88 µg/dL  30.88-49.60 µg/dL  ≥49.61 µg/dL | 1  0.72 (0.50-1.03)  1.01 (0.70-1.45) | Age, solar damage, skin type, number of prior BCCs, number of prior SCCs, BMI, treatment group, high-density lipoprotein-cholesterol, and low-density lipoprotein-cholesterol |
|  |  |  |  |  |  |  |  |  |  | <34.87 µg/dL  34.87-54.40 µg/dL  ≥54.41 µg/dL | 1  0.72 (0.50-1.03)  1.01 (0.70-1.45) |  |
|  |  |  |  |  | 85 |  |  | Squamous cell carcinoma |  | <30.88 µg/dL  30.88-49.60 µg/dL  ≥49.61 µg/dL | 1  1.06 (0.59-1.89)  0.99 (0.58-2.01) |  |
|  |  |  |  |  |  |  |  |  |  | <34.87 µg/dL  34.87-54.40 µg/dL  ≥54.41 µg/dL | 1  1.06 (0.59-1.89)  0.99 (0.58-2.01) |  |
| Kabat et al.  2009 | US,  WHI | 50-79 | F:  5450 | 12 | 190 | Serum lycopene | HPLC | Breast cancer | Self-reported | <0.30 ng/mL  0.30-<0.47 ng/mL  ≥0.47 ng/mL | 1  1.02 (0.69-1.51)  1.34 (0.92-1.94) | Age, education, ethnicity, BMI, oral contraceptive use, hormone therapy, age at menarche, age at first birth, age at menopause, alcohol, family history of breast cancer, history of breast biopsy, physical activity, energy intake, randomization status in hormone therapy, calcium plus vitamin D, and dietary modification trials |
| Kabat et al.  2012 | US,  WHI | 50-79 | F:  5477 | 12 | 88 | Serum lycopene | HPLC | Colorectal cancer | Self-reported | <0.30 ng/mL  0.30-<0.47 ng/mL  ≥0.47 ng/mL | 1  0.77 (0.46-1.31)  0.97 (0.58-1.60) | Age, BMI, waist circumference, servings of alcohol per week, physical activity, age at first live birth, ethnicity, participation in the OS or treatment arm of each of the clinical trials. |
| Karppi et al.  2009 | Finland,  KIHD | 42-60 | M:  997 | 15 | 141 | Serum lycopene | HPLC | Total cancer | Medical records | <0.08 µmol/L  0.08-0.19 µmol/L  >0.19 µmol/L | 1  0.74 (0.50-1.09)  0.55 (0.34-0.89) | Age, examination year, intake of alcohol, family history of cancer, physical activity, waist-to-hip ratio, education, years of smoking, and serum folate. |
|  |  |  |  |  |  |  |  | Prostate cancer |  | <0.08 µmol/L  0.08-0.19 µmol/L  >0.19 µmol/L | 1  1.10 (0.58-2.08)  0.78 (0.37-1.66) |  |
| Ito et al.  2002 | Japan,  CHEP | 39-80 | M/F:  2444 | 18 | 76 | Serum lycopene | HPLC | Cancer mortality | Medical records | High vs. low | 0.37 (0.19-0.72) | Gender, age, habits of smoking and alcohol consumption, and serum levels of total cholesterol and GPT activity |
| Mayne et al.  2004 | US,  NR | 20-79 | M/F:  259 | 7 | 34 | Plasma lycopene | HPLC | Cancer mortality | Reported | High vs. low | 0.65 (0.31-1.29) | Age, gender, treatment arm, time-dependent smoking, baseline plasma cholesterol, study site |
| Min et al.  2014 | US,  NHANES III | ≥20 | M/F:  10382 | 18 | 161 | Serum lycopene | HPLC | Lung Cancer mortality | Medical records | ≥29 vs. ≤13 µg/dL | 0.67 (0.42-1.07) | Age, gender, ethnicity, education, alcohol consumption, exercise, smoking status, pack-year of smoking, obesity, total cholesterol, daily fat intake and vegetable and fruit consumption |
| Shardell et al.  2011 | US,  NHANES III | ≥20 | M/F:  13293 | 18 | 645 | Serum lycopene | HPLC | Cancer mortality | Medical records | <0.29 μmol/L  0.29-0.43 μmol/L  0.44-0.58 μmol/L  >0.58 μmol/L | 1  0.96 (0.91-1.02)  0.68 (0.59-0.78)  1.14 (0.79-1.64) | Age, gender, ethnicity, marital status, education, lifestyle behaviors (alcohol consumption, smoking status, multivitamin/ multimineral use, physical activity), BMI, and other individual carotenoids, biomarkers (DBP, SBP, total cholesterol, HDL cholesterol, CRP), use of blood pressure medication, use of cholesterol-lowering medication, and comorbidities (congestive heart failure, cancer, diabetes, emphysema, stroke). |
| Pouchieu et al.  2014 | France,  SU.VI.MAX | 51.6 | M/F:  318 | 7.5 | 159 | Plasma lycopene | HPLC | Total cancer risk | Self-reported | Q4 vs. Q1 | 1.83 (0.90-3.46) | Age, intervention group, and number of dietary records, BMI, height, smoking status, physical activity, alcohol intake, educational level, family history of breast cancer, menopausal status and use of hormonal treatment for menopause at baseline, number of children, and energy, lipid, and fruit and vegetable intakes. |
|  |  | 49.8 | F:  200 |  | 100 |  |  | Breast cancer |  | Increase of 0.1 µmol/L | 1.04 (0.95-1.13) |  |

Abbreviation: BMI, body mass index; CI, confidence interval; RR, risk ratio; NR, not reported; µg/dL, microgram(s) per deciliter; n, number; y, year; US, the United States; HPLC, high-performance liquid chromatography; Q, quartile; T, tertile; vs, versus; EPIC, European Prospective Investigation into Cancer and Nutrition; F, female; M, male, HPFS, Health Professional Follow-up Study; NHS, Nurse Health Study; NHS II, Nurse Health Study II; SWHS, Shanghai Women’s Health Study; WHI, Women’s Health Initiative Study; PLCO, The Prostate, Lung, Colorectal, and Ovarian Trial, MEC, The Multiethnic Cohort Study; NHANES, National Health and Nutrition Examination Survey; SU.VI.MAX, Supplémentation en Vitamines et Minéraux Antioxydants; JPHC, Japan Public Health Center-based Prospective Study; JACC, Japan Collaborative Cohort Study study; US, the United States; ABA, Australian Blue Asbestos; CBMC, Columbia Missouri Breast Cancer; PHS, Physicians’ Health Study; CARET, Alpha Tocopherol Beta Carotene; PCPT, Prostate Cancer Prevention Trial; SCCS, Southern Community Cohort Study; CLUE, Clue Cohort Study; WHS, Women’s Health Study; CPS, Cancer Prevention Study; SCS, Shanghai Cohort Study; ISO-BCC, Isotretinoin-Basal Cell Carcinoma Prevention Trial; KIHD, Kuopio Ischaemic Heart Disease; CHEP, Contact Heat Evoked Potential; VIP, Västerbotten Intervention Program; MONICA, Monitoring Trends and Determinants in Cardiovascular Disease; MSP: Molecular Signature in Pregnancy

^1^Presented as mean or range

^2^ Presented as max or mean

**Supplementary Table 4:** Quality assessment of prospective studies included in this meta-analysis on tomato and lycopene intake with cancer risk and mortality^1^

|  | Total score | |  |  |  |  |  |  |  |  |  |
| --- | --- | --- | --- | --- | --- | --- | --- | --- | --- | --- | --- |
|  | Representativeness of the exposed cohort | Selection of the non-exposed cohort | | Ascertainment of exposure | Demonstration that outcome of interest was not present at start of study | Study controls for energy intake | Study controls for any additional factor | Assessment of outcome | Was follow-up long enough for outcomes to occur (>10 years) | Adequacy of follow up of cohorts (loss-to-follow up <20%) | **Total score** |
| Ambrosini et al. 2008 | * | * | | - | * | - | * | * | * | * | 7 |
| Cui et al. 2008 | * | * | | - | * | * | * | - | - | * | 6 |
| Cui et al. 2011 | * | * | | - | * | * | * | - | * | * | 7 |
| Fraser et al. 2020 | * | * | | - | * | * | * | - | - | * | 6 |
| Giovannucci et al. 1995 | * | * | | - | * | * | * | - | - | * | 6 |
| Han et al. 2013 | * | * | | - | * | * | * | * | * | * | 8 |
| Ho et al. 2015 | * | * | | - | * | * | * | - | * | * | 7 |
| Holick et al. 2002 | * | * | | - | * | * | * | * | * | * | 8 |
| Holick et al. 2005 | * | - | | - | * | * | * | - | * | * | 7 |
| Kim et al. 2019 | * | * | | - | * | * | * | - | * | * | 7 |
| Kirsh et al. 2006 | * | * | | * | * | * | * | * | - | * | 8 |
| Larsson et al. 2007 | * | * | | - | * | * | * | * | - | * | 7 |
| Larsson et al. 2010 | * | * | | - | * | * | * | * | - | * | 7 |
| Malila et al. 2002 | * | * | | - | * | * | * | * | - | * | 7 |
| Michaud et al. 1999 | * | * | | - | * | * | * | - | * | * | 7 |
| Michaud et al. 2000 | * | * | | - | * | * | * | - | * | * | 7 |
| Michaud et al. 2002 | * | * | | - | * | * | * | * | * | * | 8 |
| Navarro Silvera et al. 2006 | * | * | | - | * | * | * | * | * | * | 8 |
| Neuhouser et al. 2003 | * | * | | - | * | - | * | * | * | * | 7 |
| Nouraie et al. 2005 | * | * | | - | * | - | * | * | * | * | 7 |
| Pantavos et al. 2014 | - | * | | - | * | - | * | * | * | * | 6 |
| Park et al. 2009 | * | * | | - | * | * | * | * | - | * | 7 |
| Park et al. 2015 | * | * | | - | * | - | * | * | * | * | 7 |
| Schuurman et al. 2002 | * | * | | - | * | - | * | * | - | * | 6 |
| Stram et al. 2006 | * | * | | - | * | - | * | * | - | * | 6 |
| Thomson et al. 2008 | * | * | | - | * | * | * | - | - | * | 6 |
| Xu et al. 2021 | * | * | | - | * | * | * | * | * | * | 8 |
| Xu et al. 2021 | * | * | | - | * | * | * | * | * | * | 8 |
| Yuan et al. 2003 | * | * | | - | * | - | * | * | - | * | 6 |
| Zhang et al. 1999 | * | * | | - | * | * | * | - | * | * | 7 |
| Zu et al. 2014 | * | * | | - | * | * | * | - | * | * | 7 |
| Mazidi et al. 2020 | * | * | | - | * | * | * | * | * | * | 8 |
| Wang et al. 2016 | * | * | | - | * | - | * | * | * | * | 7 |
| Diallo et al. 2016 | - | * | | * | * | * | * | - | * | * | 7 |
| Dunneram et al. 2019 | * | * | | - | * | - | * | * | * | * | 7 |
| Kiani et al. 2006 | * | * | | - | * | - | * | * | * | * | 7 |
| Lin et al. 2006 | * | * | | - | * | * | * | - | * | * | 7 |
| Masala et al. 2012 | * | * | | - | * | * | * | * | * | * | 8 |
| Mills et al. 1989 | * | * | | - | * | - | * | * | - | * | 6 |
| Nothlings et al. 2006 | * | * | | - | * | * | * | * | - | * | 7 |
| Perez-Cornago et al. 2017 | * | * | | - | * | * | * | - | * | * | 7 |
| Suzuki et al. 2013 | * | * | | - | * | * | * | * | * | * | 8 |
| Takachi et al. 2010 | * | * | | - | * | - | * | * | - | * | 6 |
| Colditz et al. 1985 | - | * | | - | * | - | * | * | - | * | 5 |
| Sakauchi et al. 2004 | * | * | | - | * | - | * | * | * | * | 7 |
| Takata et al. 2013 | * | * | | * | * | * | * | - | - | * | 7 |
| Kristal et al. 2010 | - | * | | - | * | - | * | * | - | * | 5 |
| Agalliu et al. 2011 | - | * | | - | * | - | * | * | * | * | 6 |
| Botterweck et al. 2000 | - | * | | - | * | - | * | * | - | * | 5 |
| de Munter et al. 2015 | - | * | | - | * | * | * | * | * | * | 7 |
| Jain et al. 2000 | - | * | | - | * | * | * | * | * | * | 7 |
| Lane et al. 2016 | - | * | | - | * | * | * | * | * | * | 7 |
| Rohan et al. 2002 | - | * | | - | * | * | * | * | * | * | 7 |
| Steinmetz et al. 1993 | - | * | | - | * | * | * | * | - | * | 6 |
| Terry et al. 2002 | - | * | | - | * | * | * | * | * | * | 7 |
| Terry et al. 2002 | - | * | | - | * | * | * | * | * | * | 7 |
| Thomas et al. 2020 | * | * | | * | * | * | * | * | * | * | 9 |
| Voorrips et al. 2000 | - | * | | - | * | - | * | * | - | * | 5 |
| Zeegers et al. 2001 | - | * | | - | * | - | * | * | - | * | 5 |
| Sesso et al. 2005 | * | * | | - | * | - | * | * | * | * | 7 |
| Schuurman et al. 1998 | - | * | | - | * | - | * | * | - | * | 5 |
| Maasland et al. 2014 | - | * | | - | * | - | * | * | * | * | 6 |
| Boggs et al. 2010 | * | * | | - | * | * | * | - | * | * | 7 |
| Heinen et al. 2012 | - | * | | - | * | * | * | * | * | * | 7 |
| Steevens et al. 2011 | - | * | | - | * | - | * | * | * | * | 6 |
| Stolzenberg-Solomon et al. 2002 | * | * | | - | * | * | * | * | * | * | 8 |
| Koushik et al. 2006 | * | * | | - | * | * | * | - | * | * | 7 |
| Cho et al. 2003 | * | * | | - | * | * | * | - | * | * | 7 |
| Farvid et al. 2018 | * | * | | - | * | * | * | - | * | * | 7 |
| Horn-Ross et al. 2002 | * | * | | - | * | * | * | * | - | * | 7 |

**^1^**According to the Newcastle-Ottawa Scale (NOS) criteria

**Supplementary Table 5:** Quality assessment of prospective studies included in this meta-analysis on blood levels of lycopene with cancer risk and mortality ^1^

|  | Total score | |  |  |  |  |  |  |  |  |  |
| --- | --- | --- | --- | --- | --- | --- | --- | --- | --- | --- | --- |
|  | Representativeness of the exposed cohort | Selection of the non-exposed cohort | | Ascertainment of exposure | Demonstration that outcome of interest was not present at start of study | Study controls for energy intake | Study controls for any additional factor | Assessment of outcome | Was follow-up long enough for outcomes to occur (>10 years) | Adequacy of follow up of cohorts (loss-to-follow up <20%) | **Total score** |
| Bakker et al. 2016 | - | * | | * | * | * | * | * | * | * | 8 |
| Batieha et al. 1993 | - | * | | * | * | - | * | * | * | * | 7 |
| Beilby et al. 2010 | - | * | | * | * | - | * | * | * | * | 7 |
| Breslow et al. 1995 | - | * | | * | * | - | * | * | * | * | 7 |
| Dorgan et al. 1998 | - | * | | * | * | - | * | * | * | * | 7 |
| Dorjgochoo et al. 2009 | - | * | | * | * | * | * | * | * | * | 8 |
| Eliassen et al. 2015 | - | * | | * | * | - | * | * | * | * | 7 |
| Epplein et al. 2009 | - | * | | * | * | - | * | * | * | * | 7 |
| Gann et al. 1999 | - | * | | * | * | - | * | * | * | * | 7 |
| Gill et al. 2009 | - | * | | * | * | - | * | * | * | * | 7 |
| Goodman et al. 2003 | - | * | | * | * | - | * | * | * | * | 7 |
| Helzlsouer et al. 1989 | - | * | | * | * | - | * | * | * | * | 7 |
| Helzlsouer et al. 1996 | - | * | | * | * | - | * | * | * | * | 7 |
| Hsing et al. 1990 | - | * | | * | * | - | * | * | * | * | 7 |
| Huang et al. 2003 | - | * | | * | * | - | * | * | * | * | 7 |
| Hulten et al. 2001 | - | * | | * | * | - | * | * | * | * | 7 |
| Jeurnink et al. 2015 | - | * | | * | * | - | * | * | - | * | 6 |
| Key et al. 2007 | - | * | | * | * | - | * | * | * | * | 7 |
| Kristal et al. 2011 | - | * | | * | * | - | * | * | - | * | 6 |
| Lee et al. 2021 | - | * | | * | * | - | * | * | * | * | 7 |
| Leenders et al. 2014 | - | * | | * | * | - | * | * | * | * | 7 |
| Nomura et al. 1997 | - | * | | * | * | - | - | * | * | * | 6 |
| Nomura et al. 2003 | - | * | | * | * | - | * | * | * | * | 7 |
| Nordstrom et al. 2016 | - | * | | * | * | - | * | * | - | * | 6 |
| Peng et al. 2020 | - | * | | * | * | - | * | - | * | * | 6 |
| Persson et al. 2008 | - | * | | * | * | - | * | * | * | * | 7 |
| Peters et al. 2007 | - | * | | * | * | - | * | - | - | * | 5 |
| Ros et al. 2012 | - | * | | * | * | - | * | * | * | * | 7 |
| Sato et al. 2002 | - | * | | * | * | - | * | * | * | * | 7 |
| Sesso et al. 2005 | - | * | | * | * | - | * | * | - | * | 6 |
| Sisti et al. 2015 | - | * | | * | * | - | * | - | * | * | 7 |
| Wang et al. 2015 | - | * | | * | * | - | * | - | - | * | 5 |
| Wu et al. 2004 | - | * | | * | * | - | * | * | * | * | 7 |
| Yu et al. 1999 | - | * | | * | * | - | * | * | - | * | 6 |
| Yuan et al. 2004 | - | * | | * | * | - | * | * | * | * | 7 |
| Yuan et al. 2006 | - | * | | * | * | - | * | * | * | * | 7 |
| Ito et al. 2005 | - | * | | * | * | - | * | * | * | * | 7 |
| Ito et al. 2005 | - | * | | * | * | - | * | * | * | * | 7 |
| Toniolo et al. 2001 | - | * | | * | * | - | * | * | - | * | 6 |
| Jenab et al. 2006 | - | * | | * | * | * | * | * | * | * | 8 |
| Dorgan et al. 2004 | - | * | | * | * | - | * | - | - | * | 5 |
| Kabat et al. 2009 | - | * | | * | * | * | * | - | * | * | 7 |
| Kabat et al. 2012 | - | * | | * | * | - | * | - | * | * | 6 |
| Karppi et al. 2009 | - | * | | * | * | - | * | * | * | * | 7 |
| Ito et al. 2002 | - | * | | * | * | - | * | * | * | * | 7 |
| Mayne et al. 2004 | - | * | | * | * | - | * | - | - | * | 5 |
| Min et al. 2014 | * | * | | * | * | - | * | * | * | * | 8 |
| Shardell et al. 2011 | * | * | | * | * | - | * | * | * | * | 8 |
| Pouchieu et al. 2014 | - | * | | * | * | * | * | - | - | * | 6 |

**^1^**According to the Newcastle-Ottawa Scale (NOS) criteria

**Supplementary Table 6**: Summary risk estimates for the associations of tomato and lycopene with cancer risk and mortality in adults based on a random-effects model

|  |  |  |  | n^1^ | Pooled RR (95%CI)^2^ | P^3^ | I^2^ (%)^4^ | P-heterogeneity |
| --- | --- | --- | --- | --- | --- | --- | --- | --- |
| **The highest vs. lowest comparison for cancer risk** | | | | | |  |  |  |
|  | Total tomato intake | | | 19 | 0.97 (0.89-1.05) | 0.456 | 61.0 | <0.001 |
|  | Raw tomato intake | | | 5 | 1.02 (0.93-1.13) | 0.683 | 26.7 | 0.243 |
|  | Lycopene intake | | | 24 | 0.95 (0.92-0.99) | 0.024 | 26.4 | 0.117 |
|  | Lycopene levels | | | 42 | 0.88 (0.82-0.95) | <0.001 | 15.0 | 0.204 |
| **The highest vs. lowest comparison for cancer mortality** | | | | | |  |  |  |
|  | Total tomato intake | | | 4 | 0.87 (0.73-1.04) | 0.117 | 65.7 | 0.033 |
|  | Lycopene intake | | | 3 | 0.91 (0.80-1.04) | 0.151 | 86.5 | 0.001 |
|  | Lycopene levels | | | 4 | 0.67 (0.41-1.09) | 0.107 | 70.9 | 0.016 |
| **Linear dose-response association for cancer risk** | | | | | |  |  |  |
|  | Total tomato intake | | | 10 | 1.00 (0.95-1.05) | 0.919 | 55.4 | 0.017 |
|  | Raw tomato intake | | | 5 | 0.99 (0.95-1.03) | 0.573 | 0 | 0.636 |
|  | Lycopene intake | | | 22 | 0.99 (0.97-1.01) | 0.358 | 39.5 | 0.031 |
|  | Lycopene levels | | | 34 | 0.97 (0.93-1.00) | 0.071 | 99.2 | <0.001 |
| **Linear dose-response association for cancer mortality** | | | | | |  |  |  |
|  | Lycopene intake | | | 3 | 0.91 (0.78-1.07) | 0.249 | 94.6 | <0.001 |

^1^Number of effect sizes

^2^Obtained from the random-effects model

^3^ Obtained from the Q-test

^4^Inconsistency – the percentage of variation across studies due to heterogeneity
